# Catalytic Upcycling of Barrier Plastic Films by Selective Deoxygenation of Ethylene Vinyl Alcohol over Pd/MoO_3_

**DOI:** 10.1021/acssuschemeng.5c13705

**Published:** 2026-06-01

**Authors:** Samira Abdolbaghi, Laura A. Gomez, Dai-Phat Bui, Mohammad Reza Razzaghi, Kevin Nelson, Lance L. Lobban, Steven P. Crossley

**Affiliations:** † School of Sustainable Chemical, Biological and Materials Engineering, 6187 University of Oklahoma, Norman, Oklahoma 73019, United States; ‡ Amcor, Neenah Innovation Center, Neenah, Wisconsin 54956, United States

**Keywords:** polymer upcycling, plastic waste, catalytic deoxygenation, multilayer films, biphasic solvent system, reaction kinetics

## Abstract

Multilayer plastic films that combine polar barrier polymers, such as ethylene vinyl alcohol (EVOH), with nonpolar polyolefins, such as polyethylene (PE), are widely used in food packaging, but their recycling remains challenging because of phase separation during reprocessing. Here, we address this limitation through the selective deoxygenation of EVOH over a Pd-promoted molybdenum oxide (MoO_3_) catalyst, enabling C–O bond cleavage while preserving the polymer backbone. The catalytic performance was evaluated in both a monophasic γ-valerolactone (GVL) system and a biphasic GVL/decalin system. While the monophasic GVL system achieved moderate oxygen removal of up to 31.62% after 8 h, the biphasic GVL/decalin system significantly enhanced performance, reaching up to 41.1% oxygen removal in only 1 h, corresponding to a 2.4–2.8-fold increase in the reaction rate. Notably, the biphasic system enabled in situ product separation, with nonpolar deoxygenated products partitioning into the decalin phase, thereby reducing product accumulation on the catalyst surface. Applied to a commercial multilayer film containing 80 wt % LDPE and 20 wt % EVOH, the system achieved near-complete deoxygenation of 83.5% after 1 h over Pd5%/MoO_3_. Kinetic analysis revealed a positive half-order dependence on H_2_ over Pd/MoO_3_, highlighting the critical role of Pd in H_2_ dissociation and spillover, which facilitates the rate-determining step. Overall, this work establishes an efficient biphasic catalytic platform for upgrading oxygen-containing barrier layers in multilayer plastic films into more recyclable polyolefin-like materials.

## Graphical abstract

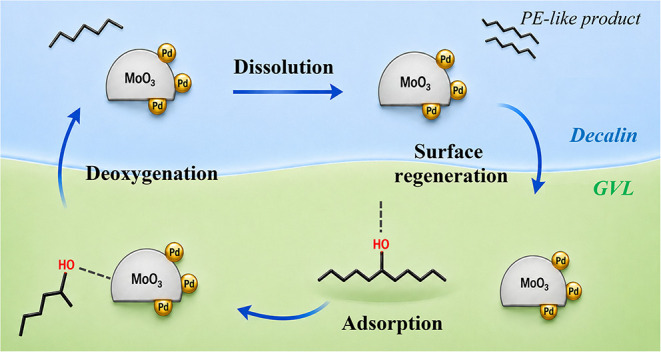

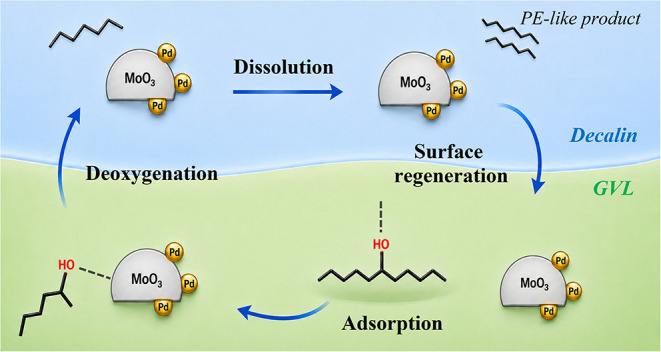

## 1 Introduction

Plastic production has increased substantially over the last few decades. It has been estimated that 464 million metric tons of plastic were produced in 2020.^1,2^ The majority of this production was discarded in landfills or incinerated, with less than 10% of plastics having been recycled.^3,4^ This annual plastics use is expected to surpass 884 Mt by 2050, resulting in environmental challenges including pollution, microplastics, and greenhouse gas emissions.^5^ These projections underscore the necessity for advanced recycling technologies to address the growing crisis of plastic waste and its environmental effects.^6^ Current plastic recycling techniques mainly include mechanical and chemical methods, each with distinct strengths and limitations. Mechanical recycling remains the most prevalent approach but is often limited by reduced material quality when dealing with contaminated or mixed plastic waste streams.^7,8^

Chemical recycling offers the capability to produce higher-value or near-virgin quality materials and is generally more effective at recycling mixed, contaminated, and multilayer plastic waste streams.^9^ However, chemical recycling of real-world contaminated plastics remains technically complex and costly due to the inherent heterogeneity and incompatibility of polymers, the presence of additives and contaminants, high energy requirements, and persistent challenges in achieving economical scale-up.^10,11^ Nevertheless, rapidly emerging approaches such as solvent-based dissolution precipitation, supercritical fluid processing, catalytic pyrolysis, hydrothermal liquefaction, and gasification^8,10,12,13^ are demonstrating enhanced commercial potential and show strong promise in addressing the limitations of recycling complex and contaminated plastic waste streams.^14–16^

Polyethylene (PE) is the most widely used plastic worldwide. It is valued for its low cost and versatile properties, which make it appropriate for a wide range of applications. PE multilayer films are widely used in food packaging, consumer, and pharmaceutical products.^17^ These films often incorporate ethylene vinyl alcohol (EVOH) as one of the layers to enhance barrier performance, significantly improving resistance to oxygen permeation, extending storage shelf life, and reducing food waste. However, the use of EVOH, a polar polymer enclosed in nonpolar PE matrices, introduces complications in the recycling process, regardless of the advantages it offers. Although EVOH commonly accounts for a small portion (5–10 wt %) of the entire laminated film composition, its presence complicates delamination and recycling.^18,19^ While mechanical recovery remains the prevailing approach for processing diverse plastic waste, it is often unsatisfactory for these laminated films due to the incompatibility of immiscible plastic layers and the presence of nonpolymeric constituents, such as aluminum and organic or inorganic coatings.^20–22^

To overcome this challenge, advanced isolation techniques are being developed to recycle distinct polymers.^23,24^ Nevertheless, when tackling mixed plastics incorporating EVOH, most research has prioritized a selective strategy to isolate EVOH. Walker et al. utilized a noncatalytic solvent-targeted recovery and precipitation (STRAP) process, an approach that can separate up to 10 common polymer layers from a multilayer film.^25,26^ These isolation approaches are effective in separating specific polymer compositions. However, they are susceptible to economic and environmental limitations due to their demand for large amounts of various solvents to enable film layer isolation.^14^ As an alternative approach, selective deoxygenation is a promising approach that involves the catalytic removal of oxygen-containing functional groups from polar polymers such as EVOH (Scheme 1). This selective deoxygenation strategy is often studied in biomass conversion to produce valuable hydrocarbons or fuel precursors.^27,28^ Employing this approach to selectively convert EVOH could enhance the compatibility of polar polymers with existing recycling systems by transforming the EVOH layer into a nonpolar PE-like layer. A limited number of studies have explored the selective deoxygenation of EVOH. Bui et al. demonstrated that EVOH could be selectively deoxygenated over Pd/TiO_2_ catalysts, attributing this activity to the formation of acid sites on the TiO_2_ surface, as well as unique active sites at the metal support.^29^

**Scheme 1:**
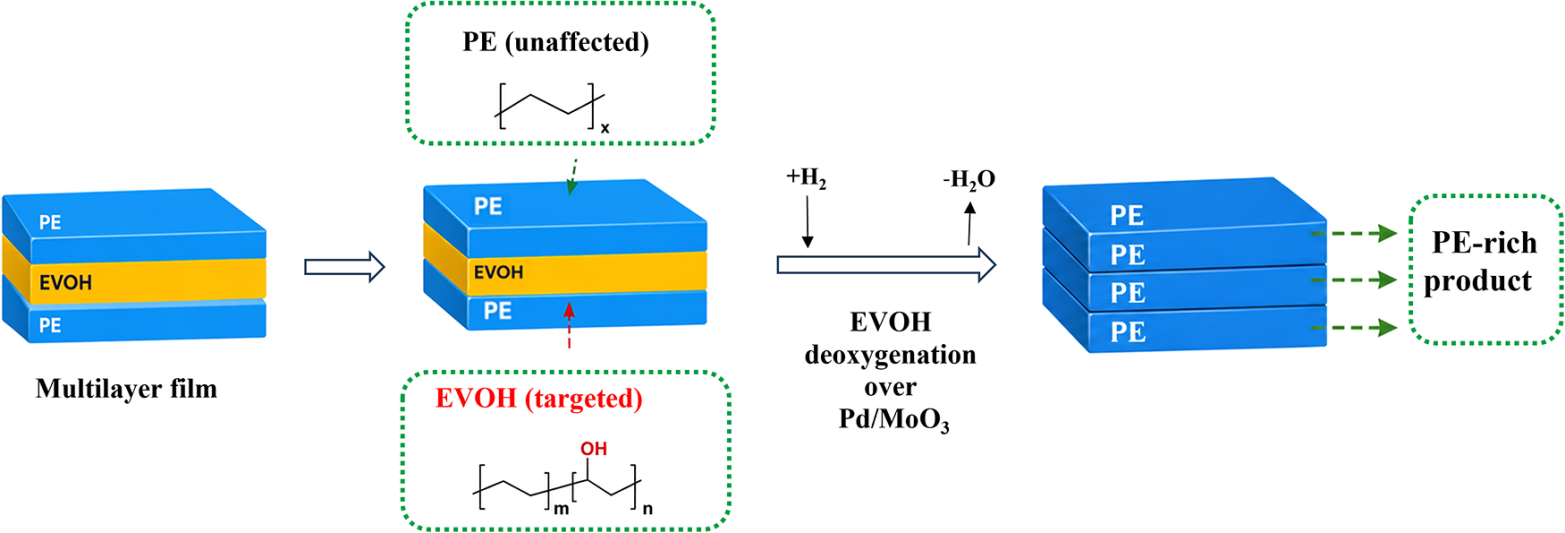
Proposed Catalytic Deoxygenation of EVOH in Multilayer Films over a Pd/MoO_3_ Catalyst

Reducible metal oxides promoted by a noble metal are promising candidates for the selective deoxygenation of oxygen-rich compounds. They target C–O bond cleavage without promoting C–C hydrogenolysis.^27,30^ The main characteristic of these catalysts is the generation of highly active sites at the metal–support interface, which are effective for deoxygenation reactions.^31–34^ Moreover, redox-active catalysts can enable hydrogen spillover, which would modify the density of oxygen vacancies and potentially contribute to reaction rates in some cases.^35–37^ Over metal oxide surfaces, in addition to the number of undercoordinated oxygen vacancies, the density of hydrogen species on the oxide surface has also been shown to impact deoxygenation rates.^38^ Recent studies have shown that dispersing low-loading Pt clusters (0.5 wt %) on a MoO_3_ support significantly enhances carboxylic acid deoxygenation rates due to elevated rates of vacancy formation and higher hydrogen coverage levels on the Mo surface.^39,40^

In this study, we employed a MoO_3_ catalyst for the selective deoxygenation of EVOH to produce PE-like products. To enhance its activity, Pd metal was incorporated onto MoO_3_ to promote the removal of hydroxyl groups from EVOH by enhancing oxygen vacancy formation/regeneration, tuning the catalyst’s acidity, and facilitating hydrogen spillover and redox chemistry of the metal oxide surface.^41–44^ The deoxygenation of EVOH is investigated in monophasic and biphasic solvent systems. Notably, 35–40% of oxygen removal is achieved by utilizing a biphasic solvent system. The introduction of a second nonpolar phase facilitates in situ separation of the deoxygenated product and reduces surface accumulation and subsequent diffusion limitations, thereby enabling improved catalyst performance. Kinetic analysis reveals that the deoxygenation of the EVOH over Pd/MoO_3_ occurs through competing mechanisms that are highly influenced by hydrogen concentration near the catalytically active sites.

## 2 Experimental Section

### 2.1 Materials

All materials used in this research were purchased from Sigma-Aldrich and used as received, unless noted otherwise. The solvents employed were dimethyl sulfoxide (DMSO, 99.9%), deuterated dimethyl sulfoxide (DMSO-*d*_6_, 99.9%, from Cambridge Isotope Laboratories, Inc.), γ-valerolactone (GVL, 99%), and decahydronaphthalene (mixture of cis + trans isomers, anhydrous, ≥99%). The catalyst precursors used in this study were molybdenum­(VI) oxide (MoO_3_, >99.5 wt %, from Alfa Aesar), Pd (NO_3_)_2_·2H_2_O (∼40% Pd basis), SiO_2_ (nanopowder, 10–20 nm particle size, Sigma-Aldrich), and ammonium molybdate tetrahydrate [(NH_4_)_6_Mo_7_O_24_·4H_2_O, 99.98% trace metals basis]. EVOH with 32 mol % ethylene content and a melting point of approximately 180 °C was used as received. For the catalytic testing of commercial multilayer films, a food packaging multilayer film supplied by Amcor was used without further purification. The composition details of the multilayered film are summarized in Table S2. High-purity gases, including H_2_ (99.999%), He (99.999%), N_2_ (99.998%), O_2_/He (5% O_2_), and air (Ultra Zero), all supplied by Airgas, were used in the reaction experiments and characterization.

### 2.2 Catalyst Synthesis

Pd/MoO_3_ catalysts with Pd loadings of 2 and 5 wt %, denoted Pd2%/MoO_3_ and Pd5%/MoO_3_, respectively, were synthesized using the incipient wetness impregnation method. Pd­(NO_3_)_2_·2H_2_O (∼40% Pd basis) was slowly added dropwise on the catalyst support to ensure uniform deposition. After impregnation, the samples were dried at 80 °C for 12 h in a temperature-controlled oven, followed by calcination at 600 °C with a ramp rate of 10 °C min^–1^ for 3 h under an airflow rate of 100 mL min^–1^. For the Pd/SiO_2_ catalyst, the same process was used to have 2 wt % of Pd on the support SiO_2_, followed by a calcination process at 350 °C for 2 h.

### 2.3 Catalyst Characterization

#### 2.3.1 Oxygen (O_2_) Pulse Reaction Experiments

Oxygen chemisorption analysis was conducted using a micropulse reactor at 350 °C to quantify oxygen vacancies. Before oxygen chemisorption, 50 mg of catalyst was reduced by flowing 60 SCCM H_2_ through a quartz tube for 30 min at 200 °C. To remove residual hydrogen species from the catalyst surface, He was subsequently flowed for 30 min at 350 °C. Finally, the catalyst bed was exposed to a 5 vol % O_2_/He gas mixture for 2 h at 350 °C to quantify the number of oxygen vacancies generated under reaction conditions. Pd2%/SiO_2_ and Pd5%/SiO_2_ underwent the same reduction treatment to quantify Pd-specific oxygen chemisorption, which was then subtracted from the total oxygen uptake measured for Pd2%/MoO_3_ and Pd5%/MoO_3_. This correction accounts for oxygen chemisorption on Pd sites and isolates the contribution from surface oxygen vacancies on the MoO_3_ support.

#### 2.3.2 Scanning Transmission Electron Microscopy (STEM)

Pd-supported MoO_3_ catalyst samples were analyzed using JEOL Grand ARM STEM operated at 300 kV using the high-angle annular dark-field (HAADF-STEM) detector. STEM analysis was performed to determine the particle size of Pd metal. A copper carbon grid coated with lacey carbon was used for sample preparation.

#### 2.3.3 Scanning Electron Microscopy–Energy-Dispersive X-ray Spectroscopy (SEM–EDS)

Elemental analysis of the catalyst samples was carried out by SEM–EDS using a Zeiss Neon FE-SEM/FIB dual-beam instrument. EDS elemental mapping was employed to evaluate the distribution of elements across the catalyst surface.

#### 2.3.4 Temperature-Programmed Reduction (TPR) and Temperature-Programmed Oxidation (TPO)

The reduction behavior of MoO_3_ and Pd/MoO_3_ catalysts was investigated using a custom-built system consisting of a 0.64-cm-outer-diameter quartz tube packed with 50 mg of catalyst. The system operated under a 5 vol % H_2_/Ar mixture at a total flow rate of 15 mL min^–1^. The temperature was ramped to 1000 °C at 10 °C min^–1^ and held for 30 min. Hydrogen consumption was monitored as a function of temperature using an online thermal conductivity detector (TCD, SRI 110). A water trap was used to condense and remove water vapor before the gas stream entered the detector.

Additionally, thermogravimetric analysis (TGA)-TPR measurements were carried out to evaluate the weight loss during the reduction process using a Netzsch STA 449F1 instrument equipped with a pin thermocouple and a high-sensitivity nanobalance. The temperature was ramped from room temperature to 400 °C at a rate of 2 °C min^–1^. Approximately 195 mg of catalyst was used for each analysis. The oxidation behavior of the spent catalysts was similarly evaluated by TGA-TPO under the same instrument configuration and catalyst loading to quantify the mass loss during oxidation. In these measurements, approximately 20 mg of spent catalyst was subjected to a temperature increase to 600 °C at 2 °C min^–1^ under 5 vol % O_2_/He at a total flow rate of 60 SCCM and held at 600 °C for 1 h.

#### 2.3.5 Brunauer–Emmett–Teller (BET) Surface Area

The specific surface area of the catalysts was determined by BET analysis using nitrogen adsorption isotherms collected on a Micromeritics ASAP 2020 instrument. Prior to measurement, approximately 2 g of each catalyst was degassed under vacuum at 300 °C for 90 min. Nitrogen adsorption–desorption isotherms were then recorded at 77 K, and the BET surface area was calculated from the adsorption data in the relative pressure range of 0.05–0.30.

#### 2.3.6 Powder X-ray Diffraction (XRD)

Powder XRD analysis was performed using a Rigaku Smart Lab X-ray diffractometer using Cu Kα radiation (λ = 1.54059 Å). Data were collected at 45 kV and 200 mA with a scan rate of 10° min^–1^. The diffraction peaks were assigned by comparison with the reference data from the MINERAL database.

### 2.4 Catalytic Reaction and Polymer Product Characterization

#### 2.4.1 TGA Study of EVOH Deoxygenation over Pd/MoO_3_

TGA was employed to study the solvent-free deoxygenation of EVOH in the presence of Pd/MoO_3_ under H_2_. Catalyst loadings of 8, 12, 16, and 20 wt % relative to the EVOH content were prepared. The temperature was ramped from 40 to 150 °C at a rate of 5 °C min^–1^ and held at 150 °C for 30 min to remove physically adsorbed H_2_O. Subsequently, the temperature increased from 150 to 200 °C at the same heating rate, followed by holding the sample at 200 °C for 1 h. Throughout this process, H_2_ was continuously flowed at 40 mL min^–1^.

#### 2.4.2 Monophasic Catalytic Reaction on EVOH

Catalytic deoxygenation of EVOH was performed in a 25 mL three-neck round-bottomed flask equipped with a Graham condenser. In a typical experiment, 10 mL of GVL was added to the flask and heated to 200 °C while stirring continuously at 200 rpm. Hydrogen gas was supplied at a steady flow rate of 60 mL min^–1^ to maintain a controlled hydrogen atmosphere during the reaction. Once the temperature reached 200 °C, 10 mg of catalyst was introduced, and the mixture was stirred for 30 min. Then, 100 mg of EVOH or multilayer film was added. The reaction was allowed to proceed under these conditions for reaction times ranging from 2 to 8 h. After completion of the reaction, the system was cooled to room temperature, and the products were recovered for further analysis, as described in Section S2. For comparison, catalytic deoxygenation of EVOH was also carried out in a 100 mL Parr reactor using 100 mg of EVOH, 10 mg of catalyst, and 10 mL of GVL at 200 °C under 450 psi H_2_ and 400 rpm stirring for the desired reaction time.

The products were dissolved in DMSO-*d*_6_ and characterized by ^1^H NMR spectroscopy using a 500 MHz JEOL spectrometer to evaluate deoxygenation. Pristine EVOH was also dissolved in 6 mL of DMSO-*d*_6_ and analyzed under identical conditions as a reference, with the corresponding ^1^H NMR spectrum shown in Figure S1. Reaction conversion was determined from the decrease in the hydroxyl-group signals relative to pristine EVOH. Details of the ^1^H NMR calculations for pristine EVOH are provided in Table S1.

#### 2.4.3 Biphasic Catalytic Reaction

Biphasic catalytic deoxygenation of EVOH and a commercial multilayer film was performed under the same reaction conditions described for the monophasic system. Typically, 5 mL of decalin as the nonpolar phase and 5 mL of GVL as the polar phase were introduced into a 50 mL three-neck round-bottomed flask equipped with a Graham condenser. The mixture was heated to 200 °C under continuous magnetic stirring at 400 rpm, while hydrogen was supplied at a flow rate of 60 mL min^–1^. Upon reaching the target temperature, 10 mg of Pd/MoO_3_ catalyst was added to the flask and allowed to mix for 30 min. Subsequently, 100 mg of EVOH or commercial multilayer film was added to initiate the reaction. After 1 h, the reaction mixture was cooled to room temperature.

For reactions involving the commercial multilayer film, a two-step dissolution procedure was employed before catalysis. A film, composed of 80 wt % low-density polyethylene (LDPE) and 20 wt % EVOH, was first contacted with 5 mL of decalin at 200 °C for 30 min. This step selectively dissolved the nonpolar PE layers. Following this, 5 mL of GVL was added to the mixture to solubilize the polar EVOH component. The catalytic reaction was then carried out under the same conditions described above for the deoxygenation of pristine EVOH.

Products from each phase were isolated according to the procedure described in Section S4 and analyzed using ^1^H NMR (500 MHz JEOL). For NMR analysis, the polar product was dissolved in DMSO-*d*_6_, while the nonpolar product was dissolved in CDCl_3_. Additional compositional details of the commercial multilayer film are provided in Table S2. The composition of the polymer products generated after reaction of the multilayer film was determined by quantitative ^1^H NMR analysis based on calibration curves, as described in Section S5.

#### 2.4.4 Gas Chromatography–Mass Spectrometry (GC–MS) Analysis of GVL

Compound identification of fresh and postreaction GVL from a blank experiment was confirmed by GC–MS using a Shimadzu 1021B system equipped with an HP-5 capillary column, 25 m × 0.25 mm.

### 2.5 Reaction Kinetics

The reaction mechanism of EVOH deoxygenation over the metal oxide catalyst was investigated by varying the EVOH concentration and H_2_ partial pressure in the biphasic system. Based on this analysis, reaction orders and turnover frequencies (TOFs) were determined, with additional kinetic plots provided in Section S11. Initial deoxygenation rates at 0 min were extrapolated from reaction rates measured at 5, 10, and 15 min. The equations used for these calculations are as follows:

1 $$\mathrm{percent of oxygen removed}\;\left(\%\right)=\frac{\mathrm{change in moles of oxygen in EVOH}\;\left(\Delta \mathrm{molOxygen}\right)}{\mathrm{moles of oxygen in pristine EVOH}\;\left(\mathrm{molOxygen}\right)}\times 100\%$$

2 $$\mathrm{rate of deoxygenation}\;\left(\mathrm{mol}/g_{\mathrm{cat}}\cdot h\right)=\frac{\mathrm{change in moles of oxygen in EVOH}\;\left(\Delta \mathrm{molOxygen}\right)}{\mathrm{mass of catalyst}\;\left(g_{\mathrm{cat}}\right)\times \mathrm{reaction time}\;\left(h\right)}$$

3 $$\mathrm{TOF per bulk Pd}\;\left(s^{-1}\right)=\frac{\Delta {\mathrm{mol}}_{\mathrm{Oxygen}}}{{\mathrm{mol}}_{\mathrm{Pd}}\times s}$$

## 3 Results and Discussion

### 3.1 Catalyst Characterization

MoO_3_ has been widely investigated as a catalyst for deoxygenation reactions; however, its limited reducibility at low temperature can restrict its catalytic performance, as indicated by TPR results in Figure S3a. To address this limitation, promoter metals such as Pd are commonly incorporated to enhance catalyst reducibility.^42^ TGA-TPR results (Figure S3b) indicate that both 2 and 5% Pd-loaded MoO_3_ catalysts exhibit approximately 24% weight loss during their reduction process. The comparable weight losses observed for the two samples suggest that the MoO_3_ catalyst has reached its maximum reducibility under the tested conditions. The formation of partially reduced Mo species, Mo^5+^ and Mo^4+^, is expected based on previous studies. Among these, Mo^5+^ species have been identified as the catalytically active species responsible for C–O bond cleavage, while Mo^4+^ is often correlated with diminished deoxygenation activity.^45^

To quantify the number of oxygen vacancies formed under reaction conditions, oxygen chemisorption was performed on both Pd2%/MoO_3_ and Pd5%/MoO_3_ samples (Table S3). Pd2%/MoO_3_ catalysts exhibited slightly higher oxygen uptake compared to Pd5%/MoO_3_, likely because higher Pd loading is expected to over-reduce the catalyst surface, promoting the formation not only of Mo^5+^ but also Mo^4+^ species. However, Mo^4+^ species typically require high energy to be fully reoxidized to Mo^6+^. Wang et al. have shown that oxidation temperature for MoO_2_ to MoO_3_ occurs above 477 °C. Therefore, since O_2_ chemisorption was conducted at 350 °C, some Mo^4+^ species may not have been fully reoxidized to Mo^6+^, thereby limiting the extent of oxygen uptake and explaining the higher oxygen uptake for the Pd2%/MoO_3_ catalyst. In contrast, Mo^5+^ species, responsible for the deoxygenation pathway, can be measured at 350 °C.^46^

Figure 1 shows HAADF-STEM images and particle-size distributions of the Pd/MoO_3_ catalysts. Pd nanoparticles are observed on the MoO_3_ support for both Pd2%/MoO_3_ and Pd5%/MoO_3_, with particle sizes distributed within a similar nanometer-scale range. Thus, the higher catalytic activity of Pd5%/MoO_3_ is not assigned to a clear decrease in the Pd particle size but is instead attributed to the greater availability of Pd sites for H_2_ activation and spillover, together with Pd/MoO_3_ interfacial oxygen-vacancy sites that promote C–O bond cleavage.^33^ Additional STEM images for both samples are provided in Figures S4 and S5.

**Figure 1:**
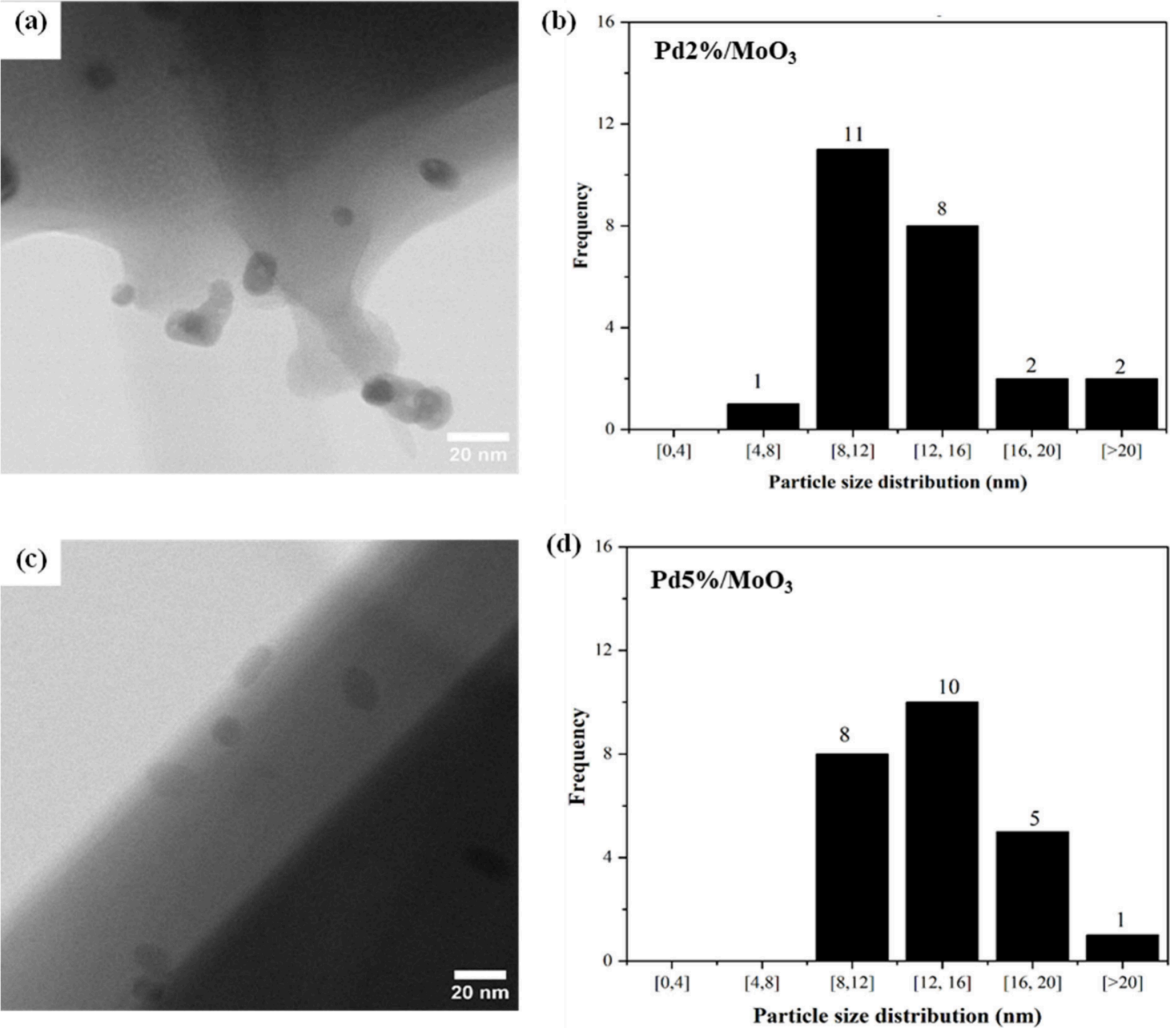
STEM images (a and c) and particle size distributions (b and d) of Pd2%/MoO_3_ and Pd5%/MoO_3_, respectively.

Table S4 summarizes the BET surface areas for the reduced MoO_3_ support and Pd-containing catalyst, determined by nitrogen physisorption. Reduced MoO_3_ exhibited a low surface area of 2.01 m^2^ g^–1^, which increased to 3.80 m^2^ g^–1^ upon addition of 2 wt % Pd and further to 6.45 m^2^ g^–1^ with 5 wt % Pd. As expected for MoO_3_-based materials reduced under mild conditions, the presence of Pd further enhances the surface area, consistent with other reports in the literature.^47^ This observation suggests that Pd loading modifies not only the hydrogen coverage but also the accessible surface area available for reaction. Specifically, the increase in surface area with Pd loading indicates that Pd incorporation modifies the accessible texture of the reduced MoO_3_ catalyst, which may increase the number of surface sites available for hydrogen activation, spillover, and reaction. These findings indicate a moderate structural modification upon Pd incorporation, which could be beneficial for catalytic performance.

SEM–EDS analysis was performed to evaluate the elemental composition and Pd distribution in the Pd2%/MoO_3_ and Pd5%/MoO_3_ catalysts. The EDS spectra (Figures S6a and S7a) confirm the expected Pd loadings of 2 and 5 wt %, respectively. Elemental mapping of Mo, O, and Pd (Figures S6 and S7) reveals a uniform dispersion of Pd across the MoO_3_ support for both catalysts.

Figure S8 presents the XRD patterns of fresh MoO_3_, MoO_3_ calcined at 600 °C, fresh Pd/MoO_3_, spent Pd/MoO_3_, and MoO_2_. The diffraction patterns of fresh MoO_3_, calcined MoO_3_, fresh Pd/MoO_3_, and spent Pd/MoO_3_ were highly similar and remained distinct from that of MoO_2_, suggesting that the MoO_3_ support preserved its bulk crystalline phase during calcination and under the reaction conditions, with no detectable conversion to MoO_2_.

TGA-TPO analysis of the spent Pd2%/MoO_3_ and Pd5%/MoO_3_ catalysts further revealed only small mass losses of approximately 0.4 and 0.5 wt %, respectively (Figure S9), suggesting that carbonaceous deposition on the catalyst surface was minimal under the applied reaction conditions.

### 3.2 EVOH Deoxygenation over Pd/MoO_3_ in Different Reaction Systems

#### 3.2.1 EVOH Deoxygenation under Solventless Conditions

TGA experiments were performed to evaluate EVOH deoxygenation over Pd/MoO_3_ under solventless conditions, establishing a baseline for comparison with the solvent-based reaction systems. As shown in Figures 2 and S10, pristine EVOH exhibited thermal stability up to 200 °C, with negligible weight loss. In contrast, the incorporation of Pd2%/MoO_3_ catalysts at loadings between 8 and 20 wt % resulted in weight losses of 0.9–5.1 wt %, corresponding to 2.74–16.06% hydroxyl-group removal (Figure 2a). Higher conversions were achieved with Pd5%/MoO_3,_ which exhibited weight losses of 1.3–6.3 wt %, equivalent to 4.19–20.45% hydroxyl-group removal. These findings demonstrate that Pd/MoO_3_ facilitates catalytic C–O bond scission in molten EVOH even in the absence of solvent. Establishing this solventless baseline enables direct differentiation between intrinsic catalytic activity and solvent-mediated effects in monophasic and biphasic systems, where enhanced mass transfer, hydrogen availability, and product removal collectively contribute to improved conversion.

**Figure 2:**
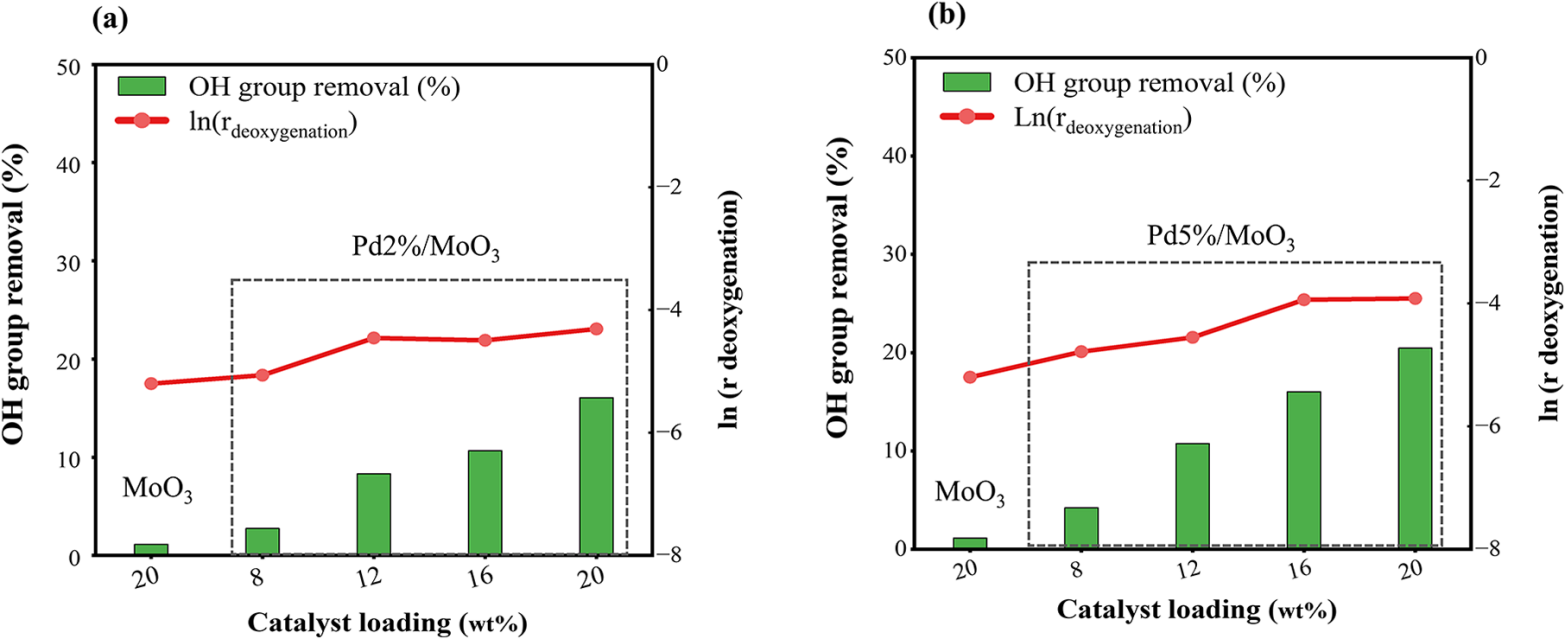
Hydroxyl-group removal (%) from EVOH as a function of the catalyst loading during solventless deoxygenation by TGA over (a) Pd2%/MoO_3_ and (b) Pd5%/MoO_3_. Reaction conditions: EVOH = 20 mg, catalyst loading = 8–20 wt % relative to EVOH, *F*_H_2__ = 40 mL min^–1^, and *T* = 200 °C.

#### 3.2.2 EVOH Deoxygenation in a Monophasic Catalytic System

To mitigate potential diffusion limitations, we have previously demonstrated that the addition of GVL as a solvent can reduce viscosity while also enhancing hydrogen flux to the active sites.^29^ EVOH deoxygenation over Pd/MoO_3_ in GVL was conducted, as shown in Scheme 2. The reaction was monitored over time to assess the evolution of deoxygenation activity. The isolated products were characterized by ^1^H NMR spectroscopy and compared with pristine EVOH (Table S1 and Figure S1). The ^1^H NMR spectrum of pristine EVOH showed characteristic chemical shifts corresponding to R–CH_3_, R–CH_2_–, and R–C­(OH)– groups, consistent with a composition of approximately 32 mol % ethylene. Figure S12 shows the ^1^H NMR spectra of the reaction products in DMSO-*d*_6_. As the deoxygenation reaction progressed over both Pd2%/MoO_3_ and Pd5%/MoO_3_, the intensity of the R-C­(OH)- signal decreased, while that of the R–CH_2_– signal increased, indicative of C–O bond cleavage and hydrogenation events.

**Scheme 2:**
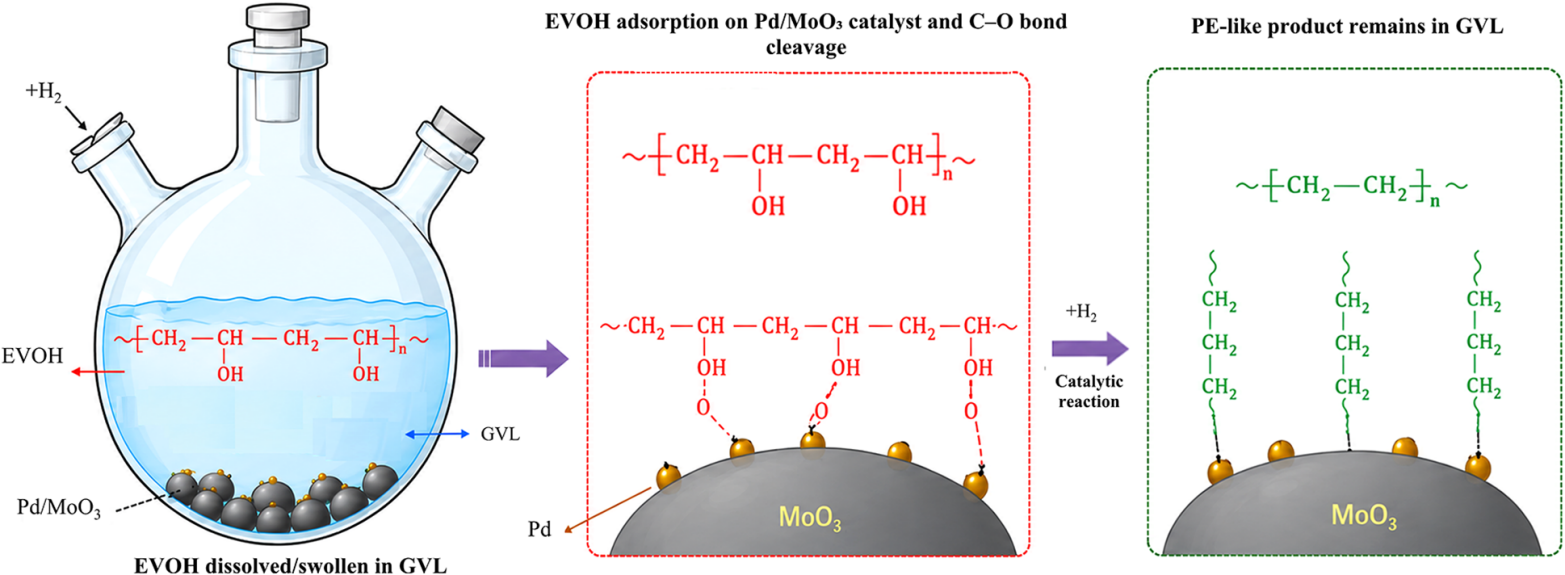
Schematic Representation of EVOH Deoxygenation over Pd/MoO_3_ in a Monophasic Reaction System

Parts a and b of Figure 3 provide a detailed analysis of the ^1^H NMR data obtained from EVOH deoxygenation reactions over Pd2%/MoO_3_ and Pd5%/MoO_3_. For Pd2%/MoO_3_, oxygen removal increased with the reaction time, from 15.45% after 2 h to 22.24% after 8 h. This corresponds to a decrease in the R–CH­(OH)– group intensity to 25.8% and an increase in R–CH_2_– species to 73.98% within the polymer chains (Figure 3a). In comparison, Pd5%/MoO_3_ catalyst demonstrates enhanced EVOH deoxygenation performance, achieving 23.1% and 31.62% oxygen removal after 1 and 8 h, respectively. These results reveal that the higher Pd loadings accelerate deoxygenation rates, as evidenced by an increase in the R–CH_2_ content from 66.3% in pristine EVOH to 76.9%, accompanied by a decrease in the R–OH content from 33.2% to 21.7% after 8 h.

**Figure 3:**
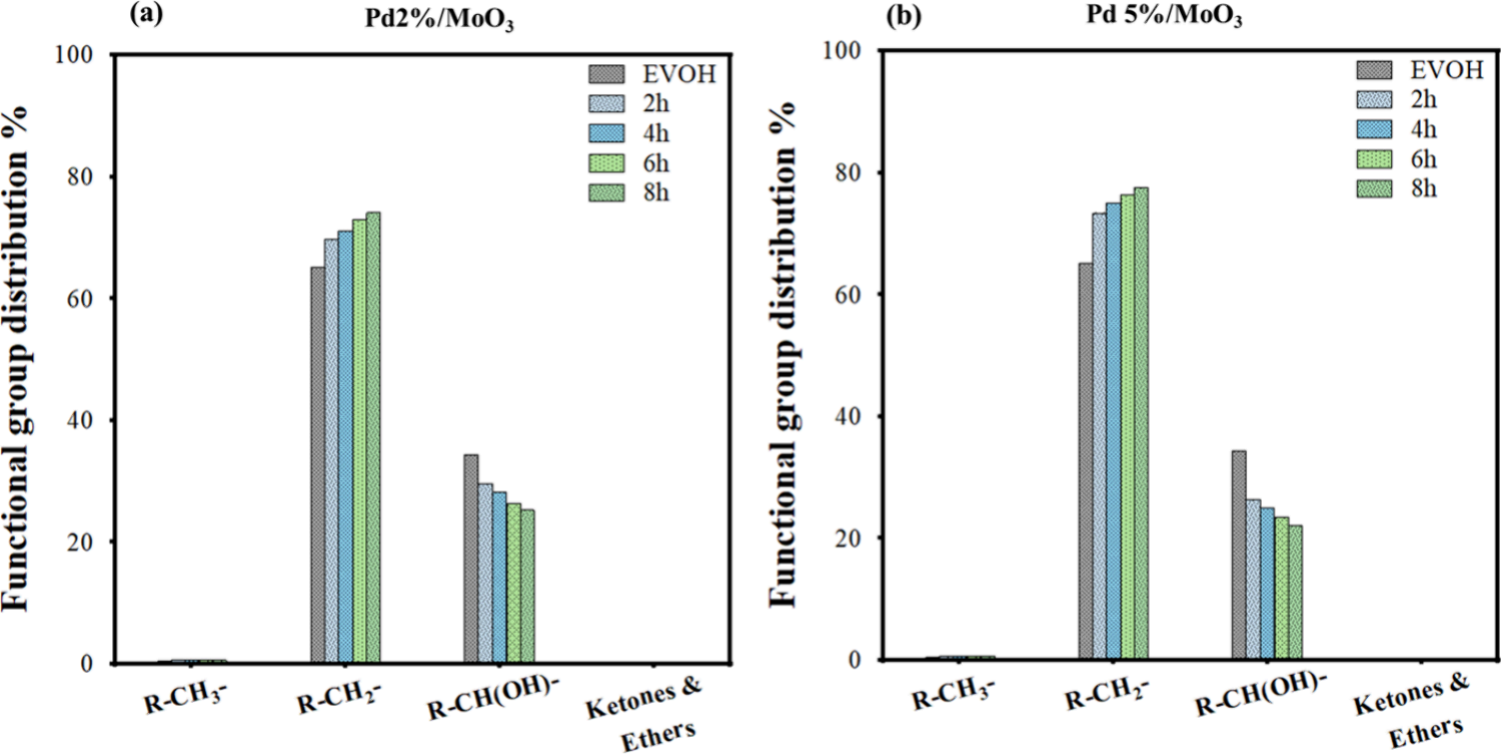
Functional-group distribution (%) of EVOH after reaction in a monophasic system using (a) Pd2%/MoO_3_ and (b) Pd5%/MoO_3_. Reaction conditions: EVOH = 100 mg, catalyst = 10 mg, *F*_H_2__ = 60 mL min^–1^, *T* = 200 °C, and GVL = 10 mL.

To assess the role of metal–support interactions in the selective C–O bond cleavage of EVOH, a comparative analysis was conducted in the monophasic system using Pd/SiO_2_ and MoO_3_ catalysts. As shown in Figure S13, both Pd/SiO_2_ and MoO_3_ exhibited negligible conversion of EVOH. This weak performance highlights the unique catalytic properties of Pd/MoO_3_, which can be attributed to the activity of the interfacial sites at the Pd/MoO_3_ interface. In contrast to the TGA-based EVOH deoxygenation experiments under flowing H_2_ at isothermal temperature, the reaction performed in the presence of GVL showed significantly suppressed formation of undesirable ketone side products, with residual ketone content limited to 0.4% and 0.6% after 8 h for the Pd2%/MoO_3_ and Pd5%/MoO_3_ catalysts, respectively. These results suggest a strong preference toward the deoxygenation and hydrogenation pathway over ketone formation in the presence of solvent. Similar trends have been observed for Pd/TiO_2_ based catalysts in monophasic reaction systems; however, MoO_3_ appears to exhibit even lower selectivity toward ketone formation.^48^

Overall, we hypothesize that the catalytic transformation of EVOH over Pd/MoO_3_ proceeds through multiple reaction pathways, as illustrated in Figure 4b. The primary pathway involves EVOH adsorption at oxygen vacancies located near the Pd/MoO_3_ interface, where coordination of hydroxyl groups promotes selective C–OH bond cleavage. This is followed by hydrogenation of the R–CH_2_–CH– intermediate to form a PE-like (R–CH_2_–CH_2_−) polymer in the final products. Competing side reactions, such as keto–enol tautomerization leading to ketone formation, may occur due to partial deoxygenation or C–O bond rearrangement.^48,49^ The deoxygenation efficiency, up to 31.62% oxygen removal, and suppression of ketone formation at both Pd loadings are attributed to the strong interfacial synergy between Pd and MoO_3_, efficient hydrogen dissociation on Pd sites and selective deoxygenation at highly active sites at the Pd/MoO_3_ interface, and the presence of abundant oxygen vacancies that promote deoxygenation and hydrogenation over side reactions. An alternative explanation consists of deoxygenation on MoO_3–*x*_ sites to yield alkenes, followed by hydrogenation over the metal catalyst to minimize the aforementioned side reactions that lead to deactivation. These findings demonstrate that optimizing Pd loading is crucial for enhancing selectivity toward PE-like products while minimizing ketone byproducts.

**Figure 4:**
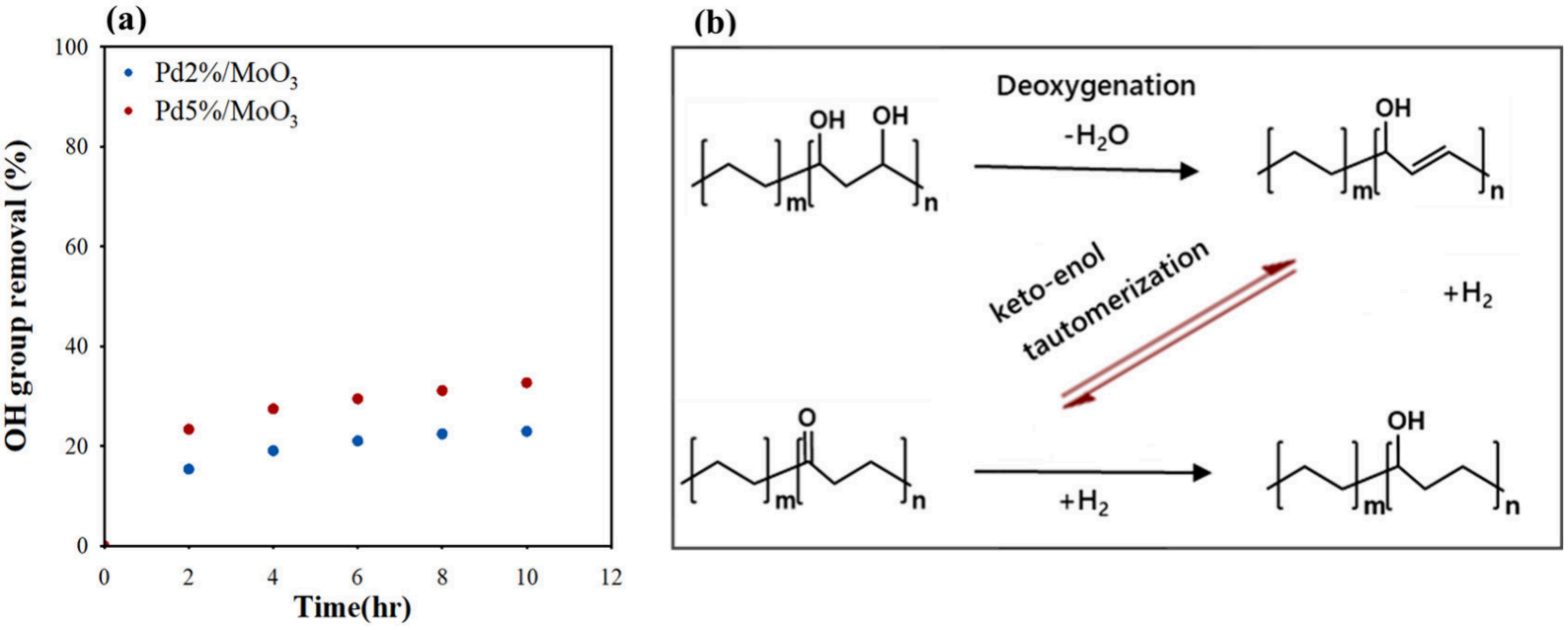
(a) Percentage of OH-group removal as a function of the reaction time in the monophasic system. Reaction conditions: EVOH = 100 mg, catalyst = 10 mg, *F*_H_2__ = 60 mL min^–1^, *T* = 200 °C, and GVL = 10 mL. (b) Proposed pathways for EVOH deoxygenation over Pd/MoO_3_.

Time course monitoring of EVOH deoxygenation in a monophasic reaction system reveals that the conversions plateau after approximately 8 h. As shown in Figure 4a, extending the reaction time beyond this point results in negligible additional hydroxyl-group removal. Similar plateau behavior was observed for both catalysts when the monophasic reaction was performed in the Parr reactor under H_2_ pressure (Figure S14), further supporting that this trend is intrinsic to the monophasic system. This observation can be attributed to several factors. The accumulation of water, a byproduct of the deoxygenation process, in the batch system may inhibit the reaction by modifying the catalyst surface or its surface properties or competing for active sites. A more likely explanation is the buildup of deoxygenated products, such as PE-like species, on the Pd/MoO_3_ catalyst surface, which could hinder access of reactants to active sites. To address this challenge, the addition of a nonpolar solvent was investigated, as discussed in the following section, to facilitate in situ separation of nonpolar products during the reaction, thereby mitigating product accumulation and enhancing catalytic efficiency.

#### 3.2.3 EVOH Conversion in a Biphasic Catalytic System

A biphasic catalytic system, consisting of two immiscible liquid phases, offers several advantages in catalytic reactions. These include simplified product isolation based on solubility differences and enhanced reaction selectivity, as products may form in one solvent phase and rapidly partition into the other. We previously demonstrated how solid catalysts localize at the interface between these two phases, increasing the interfacial area and thereby enhancing reaction rates.^50^ Motivated by these advantages, we investigated EVOH deoxygenation in a biphasic system as a strategy to overcome limitations associated with monophasic systems, particularly product accumulation on the catalyst surface that inhibits conversion. Given the polar nature of EVOH and the nonpolar nature of the resulting PE-like product, GVL was selected as the polar phase due to its miscibility with molten EVOH, while decalin was employed as the nonpolar phase, compatible with the reaction products. This solvent pairing enables efficient phase separation, facilitates in situ product removal, and streamlines downstream product isolation.

The stability of GVL under the reaction conditions was verified using blank experiments containing GVL and the catalyst. As shown in Figure S15, ^1^H NMR analysis of the postreaction solvent showed no new signals or noticeable peak shifts relative to fresh GVL, indicating no detectable solvent degradation. This conclusion was further supported by the negligible change in liquid mass before and after reaction, suggesting that GVL was neither consumed nor converted into volatile products. In addition, GC–MS analysis of fresh and postreaction GVL from the monophasic blank experiment produced nearly identical chromatograms, with no new peaks attributable to ring-opened or hydrogenolysis-derived species (Figure S16). Although previous reports have shown that GVL can undergo ring opening followed by hydrogenolysis under more severe catalytic hydrogenation conditions,^51^ no evidence of such transformations was observed under the reaction conditions employed in this study.

Figure 5 presents a comprehensive comparison of the products obtained from EVOH deoxygenation over Pd/MoO_3_ catalysts with 2 and 5% Pd loadings. The reactions were conducted in a biphasic GVL/decalin system at 200 °C under a hydrogen flow rate of 60 mL min^–1^. For reference, results from monophasic reactions conducted in pure GVL and pure decalin are also included. Among all systems, reactions in pure decalin showed the lowest activity for both catalysts, with negligible changes in the hydroxyl-group content. In contrast, monophasic reactions in GVL required 8 h to achieve moderate deoxygenation efficiencies of 22.24% and 31.62% for Pd2%/MoO_3_ and Pd5%/MoO_3_, respectively. Notably, the biphasic system exhibited much faster and more effective deoxygenation. After only 1 h, the deoxygenation efficiency reached 34.1% over Pd2%/MoO_3_ and 41.02% over Pd5%/MoO_3_, exceeding or matching the values obtained in monophasic GVL after 8 h. These results clearly demonstrate the superiority of the biphasic system in accelerating EVOH deoxygenation. The ^1^H NMR results shown in Figure 5b further support this trend. In the biphasic system, the content of R–OH groups decreased from 33.2% in neat EVOH to 20.94% and 18.07% for Pd2%/MoO_3_ and Pd5%/MoO_3_, respectively, with a corresponding increase in R–CH_2_ content from 66.4% to 79.42% and 81.1%. Meanwhile, the formation of methyl groups, ethers, and ketones remained very low, indicating that the biphasic system promotes selective C–O bond cleavage while largely preserving the polymer backbone.

**Figure 5:**
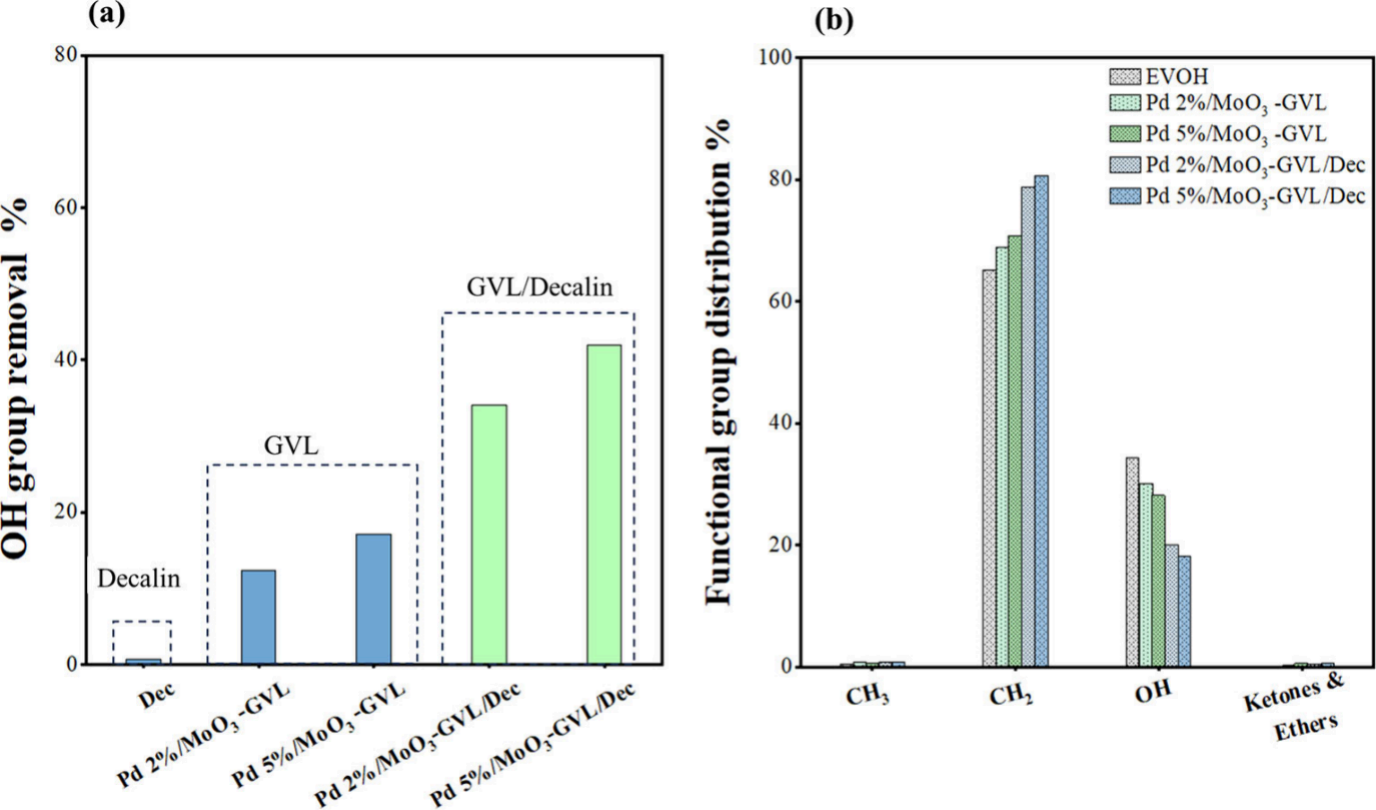
(a) Comparison of the deoxygenation efficiencies over Pd2%/MoO_3_ and Pd5%/MoO_3_ in decalin, GVL, and biphasic GVL/decalin solvent systems. (b) Functional-group distribution of EVOH products obtained in monophasic GVL and biphasic GVL/decalin systems. Reaction conditions: EVOH = 100 mg, catalyst = 10 mg, *F*_H_2__ = 60 mL min^–1^, *T* = 200 °C, *t* = 1 h, and solvent system = 5 mL of GVL + 5 mL of decalin.

Overall, these results indicate that the biphasic reaction system enhances deoxygenation efficiency, likely due to improved product removal and more effective catalyst surface regeneration, thereby mitigating diffusion limitations inherent in monophasic media. We hypothesize that the reaction begins with the adsorption of polar EVOH onto the catalyst surface in the GVL phase, where deoxygenation is initiated. As the reaction proceeds, nonpolar PE-like molecules form on the catalyst surface. Due to their immiscibility with GVL, these nonpolar products naturally migrate toward the decalin phase. This migration enables in situ product separation, continuously removing products from the catalyst surface, facilitating active-site regeneration, and maintaining catalyst activity throughout the reaction as illustrated in Scheme 3.

**Scheme 3:**
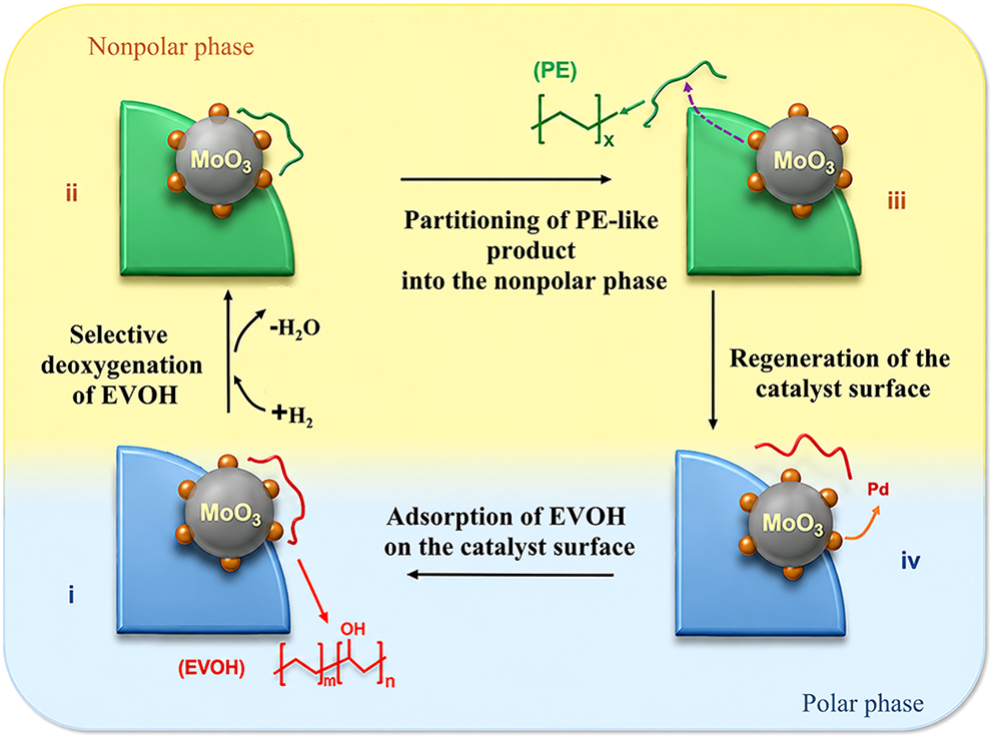
Proposed Approach for EVOH Deoxygenation over Pd/MoO_3_ in a Biphasic GVL/Decalin System

#### 3.2.4 Conversion of Commercial Multilayer Film into a PE-Rich Stream

Following the promising results obtained for EVOH deoxygenation in different solvent systems, the study was extended to a commercial multilayer film containing PE and EVOH under reaction conditions similar to those used for EVOH deoxygenation. Figure 6a shows the initial biphasic reaction mixture, with two distinct phases corresponding to the polar and nonpolar components. After reaction, a third phase, appearing as a stable emulsion interphase layer, was observed (Figure 6b), suggesting that the catalyst promotes the stabilization of more interfacial areas in proximity to the active catalysts, which may enhance mass transfer and improve catalytic turnover rates.

**Figure 6:**
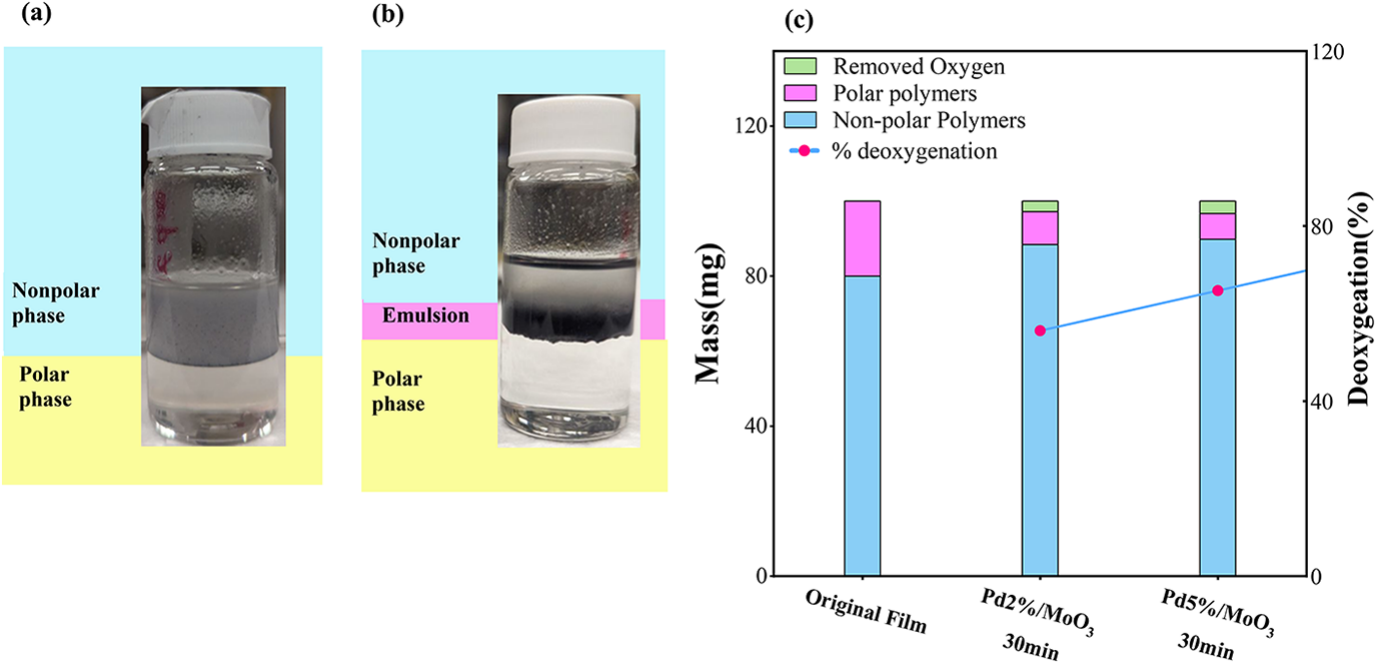
(a) Dissolved commercial multilayer film in the biphasic reaction mixture. (b) Reaction mixture after catalysis, showing the formation of an interfacial emulsion layer. (c) Product mass balance illustrating the ratio of polar to nonpolar components. Reaction conditions: film = 100 mg, Catalyst = 10 mg, F_H2_ = 60 mL min^–1^, *T* = 200 °C, *t* = 30 min, solvent system = 5 mL GVL + 5 mL decalin.

To evaluate the compositional changes after reaction and identify the products formed over both catalysts, the initial film components and the resulting reaction products were analyzed by ^1^H NMR spectroscopy. Figures S17 and S18 show the ^1^H NMR spectra of the products obtained after reaction over Pd2%/MoO_3_ and Pd5%/MoO_3_, respectively, with panel a corresponding to the polar fraction and panel b corresponding to the nonpolar fraction. The quantitative results derived from these analyses are summarized in Table S5, including the calculated EVOH content, PE content, and oxygen removal after reaction for each catalyst. From these data, a product mass balance was established by quantifying the relative amounts of the remaining polar and nonpolar fractions in the reacted film mixtures. Figure 6c demonstrates an inverse correlation between the increase in nonpolar polymer content and the corresponding decrease in polar polymer content within the film. Accordingly, an increase in CH_2_ functional groups was observed for products obtained over both Pd2%/MoO_3_ and Pd5%/MoO_3_ catalysts, consistent with NMR-derived deoxygenation efficiencies of 56.80% and 65.29%, respectively, indicating effective deoxygenation of the commercial film. Further details of the calibration procedure are provided in Section S5.

A comparative analysis shows that, under biphasic reaction conditions, the multilayer film undergoes deoxygenation more effectively than pure EVOH, as shown in Figure 7a. Pure EVOH reached only 34.1% oxygen removal over Pd2%/MoO_3_ and 41.02% over Pd5%/MoO_3_ after 1 h. By contrast, the multilayer film achieved higher oxygen removal of 56.80% and 65.29% after 30 min with the same catalysts. This enhanced performance may result from the thin-film geometry, which can improve dissolution and access of the EVOH layer to the catalyst surface, or from the tie layers present in the film, which may serve as interfacial stabilizers, thereby improving reactant access to the catalyst surface. Extending the reaction to 1 h further increased deoxygenation to 83.5% for Pd5%/MoO_3_, as shown in Figure 7b. Recent work by Bui et al. investigated the deoxygenation of commercial multilayer films using Pd/TiO_2_ based catalysts, achieving 66.1% oxygen removal after 1 h (Figure S19).^52^ In contrast, Pd5%/MoO_3_ achieved 65.29% deoxygenation after only 30 min and reached near-complete oxygen removal of 83.5% within 1 h without requiring decalin replacement. This represents a substantial improvement in both catalytic efficiency and operational practicality, eliminating constant solvent replacement and offering a more effective and scalable approach for catalytic upcycling of multilayer films. One explanation for this behavior could be due to either the differing affinity of the PE-like product for the MoO_3_ surface or the intrinsic proximity of the MoO_3_-based catalyst to the polar/nonpolar interface, both of which may facilitate PE-like product removal and enable further EVOH turnover.

**Figure 7:**
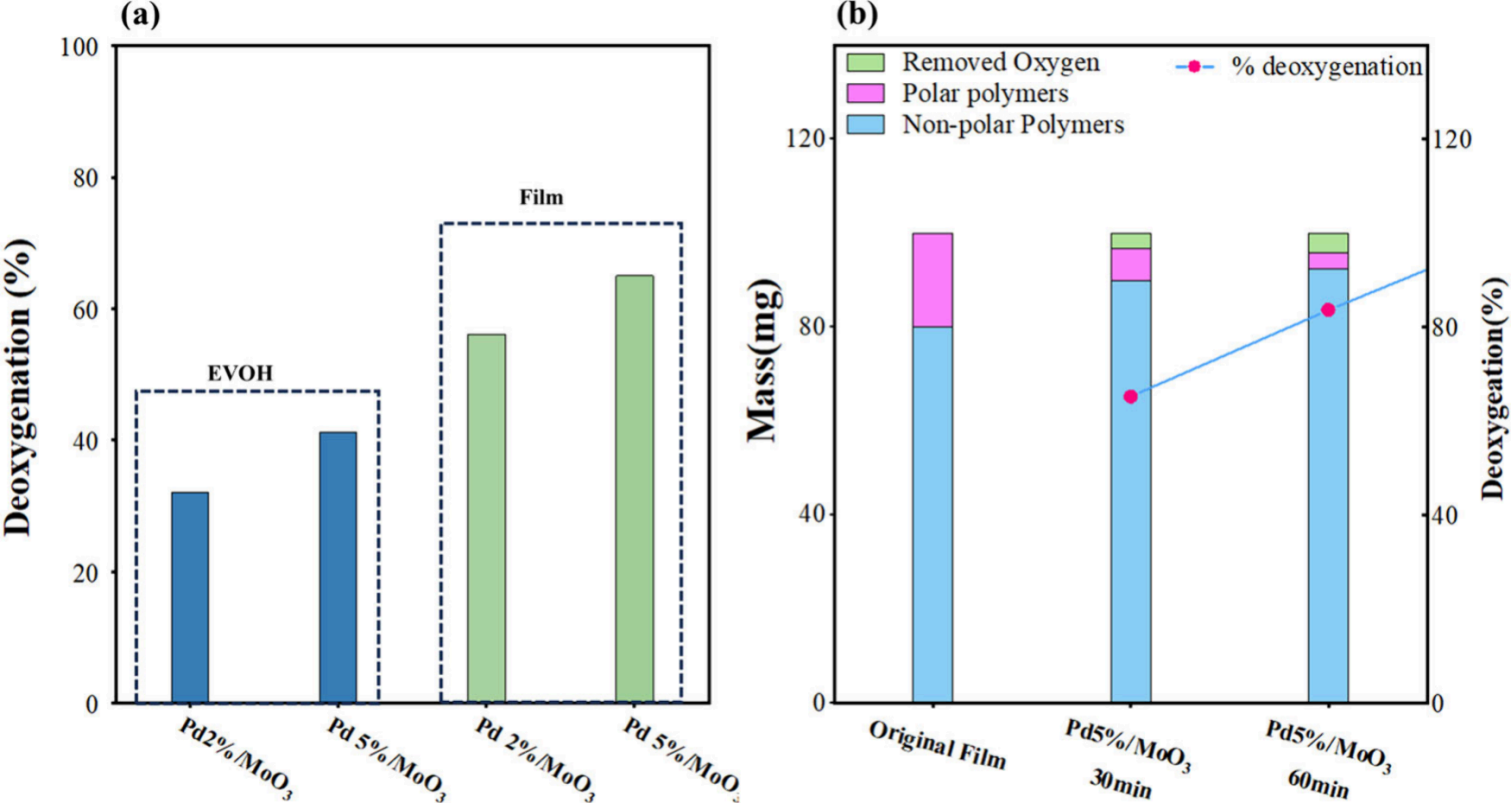
(a) Comparison of oxygen removal from pure EVOH and commercial multilayer film in a biphasic system. Reaction conditions: film = 100 mg, EVOH = 100 mg, catalyst = 10 mg, *F*_H_2__ = 60 mL min^–1^, *T* = 200 °C, reaction time = 30 min for film vs 1 h for EVOH, and solvent system = 5 mL of GVL + 5 mL of decalin. (b) Mass balance of polar and nonpolar components obtained by extending the reaction time. Reaction conditions: film = 100 mg, catalyst = 10 mg, *F*_H_2__ = 60 mL min^–1^, *T* = 200 °C, and solvent system = 5 mL of GVL + 5 mL of decalin.

From a sustainability perspective, the merits of the biphasic catalytic deoxygenation strategy can be evaluated in terms of product value, process integration, and auxiliary material use. A key barrier to multilayer film recycling is the incompatibility between polar EVOH and nonpolar PE, which leads to phase separation during reprocessing. In this approach, selective deoxygenation transforms EVOH into a PE-like material, resulting in a recycled stream with improved compositional compatibility. In contrast to solvent-based separation methods such as STRAP, which require dilute EVOH separation from the polar solvent and additional energy input and costs, the biphasic GVL/decalin system combines reaction and product partitioning in a single process. Our prior results indicate that the decalin solvent may also not be necessary in a practical system, which could further improve process simplicity.^52^ The biphasic solvent system also plays multiple functional roles within the process. Specifically, GVL acts as the polar phase that dissolves EVOH and promotes contact with the catalyst while creating a reduced viscosity environment to enable hydrogen transport, whereas the organic phase facilitates product recovery. At the same time, practical implementation will require further attention to solvent reuse, the energy demands of product and solvent recovery, and tolerance to impurities such as tie layers, adhesives, and inks present in real multilayer films.

#### 3.2.5 Kinetic Insights for EVOH Deoxygenation over Pd/MoO_3_

EVOH deoxygenation rates over Pd/MoO_3_ under flowing H_2_ were investigated to evaluate the interaction of EVOH with catalyst active sites. As shown in Figure 8a, the reaction rate increases linearly with initial EVOH concentration, indicating first-order kinetics with respect to EVOH. This finding suggests that the rate-limiting step is governed by EVOH concentration, where increased EVOH adsorption facilitates C–O bond scission and accelerates the overall reaction, without reaching full site saturation under the conditions studied. As depicted in Figure S20, increasing the catalyst loading to 20 mg resulted in an approximately 2-fold increase in oxygen removal while maintaining the same reaction order, further supporting the linear dependence on EVOH concentration.

**Figure 8:**
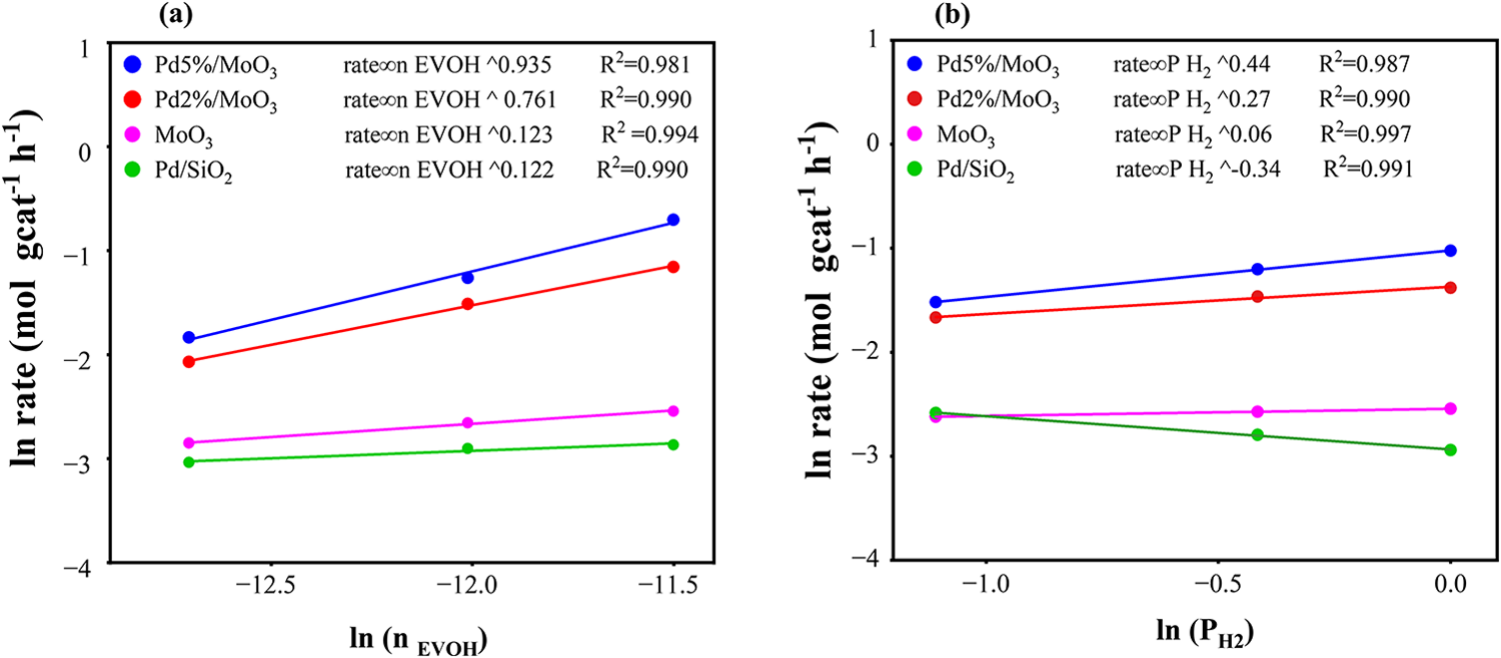
Reaction orders over Pd/MoO_3_ and Pd/SiO_2_ with respect to (a) EVOH concentration and (b) H_2_ partial pressure. Reaction conditions for EVOH concentration studies: EVOH = 30, 60, and 100 mg, P_H_2_ = 0.6 atm, F_total = 60 mL min^–1^, catalyst = 10 mg, and *T* = 200 °C. Reaction conditions for H_2_ partial-pressure studies: EVOH = 100 mg, P_H_2_ = 0.33, 0.66, and 1 atm, F_total = 60 mL min^–1^, catalyst = 10 mg, and *T* = 200 °C.

A positive rate order with respect to EVOH was also observed for MoO_3_ and Pd/SiO_2_ catalysts (Figure 8). The enhanced activity of Pd-promoted MoO_3_ is attributed to both improved H_2_ dissociation and accelerated oxygen-vacancy formation near the metal–support interface, indicating the critical role of support interactions in EVOH deoxygenation.^53^ Corresponding reaction rates and TOFs with respect to EVOH for Pd/MoO_3_ are presented in Figures S21 and S22, confirming that synergistic interactions between Pd and MoO_3_ enhance the catalytic activity.^54^

Hydrogen plays a critical role in the kinetics of EVOH deoxygenation over Pd/MoO_3_ catalysts, primarily by participating in redox chemistry that facilitates oxygen removal at the metal–support interface. Hydrogen dissociates over Pd metal sites, producing atomic hydrogen that spills over to the MoO_3_ support.^55^ This spillover hydrogen promotes the formation and regeneration of oxygen vacancies, which are essential for activating C–OH bonds in EVOH and maintaining a continuous supply of reactive hydrogen species for deoxygenation. This spillover pathway helps maintain a steady flow of hydrogen atoms, which promotes efficient deoxygenation.^56,57^

The kinetic relevance of a spillover-induced mechanism can be assessed using reaction orders with respect to hydrogen. As discussed above, the TPR results indicate that MoO_3_ reduction, even in the presence of Pd, occurs at significant rates only at temperatures above the reaction conditions. This suggests that long-range spillover followed by extensive oxygen removal from the support is unlikely under steady-state reaction conditions. Nonetheless, it is well-known that oxygen atoms in the vicinity of metal particles can be removed at higher rates, and it is not uncommon for undercoordinated support sites to reside in proximity to supported metal clusters.^58^ In other words, kinetically relevant spillover to sites near metal clusters can still occur and must be evaluated through kinetic analysis.^59^

Regardless of the overall extent of support reduction, an important mechanistic question remains, does the oxygenated polymer interact primarily with undercoordinated support sites, or does it form bonds directly with the metal during the kinetically relevant step. A spillover-induced mechanism implies that H_2_ dissociates on the Pd and spills over to generate active sites on the support, even if those sites are located near the metal–support interface. In this case, dissociated hydrogen atoms participate in product formation without requiring direct polymer–metal contact.^59^ In contrast, an interfacial mechanism requires direct binding of the polymer at metal–support interface sites. Such a mechanism, as previously reported over TiO_2_-supported catalysts, would involve carbon binding to the metal surface and C–O bond weakening through interaction with the support.^60,61^ This pathway may require initial dehydrogenation to form a C–metal bond and could therefore exhibit an inverse H_2_ dependence, depending on the number of C–H bonds cleaved before the rate-determining step.^62,63^

Our results reveal that the reaction order with respect to H_2_ in EVOH deoxygenation over MoO_3_ is approximately 0.06, indicating a near-zero-order dependence (Figure 8b). This near-zero kinetic dependence signifies that MoO_3_ alone is not effective for hydrogen dissociation under the reaction conditions. Similar behavior has been reported for TiO_2_-based systems, where limited reducibility restricts the role of hydrogen.^52^

In contrast, Pd incorporation significantly modified the reaction kinetics, increasing the H_2_ reaction order to approximately 0.50. This stronger H_2_ dependence reflects the direct involvement of H_2_-derived species in the rate-determining step for EVOH deoxygenation. The synergistic interaction between Pd and MoO_3_ enhances the overall reaction rate by coupling Pd-mediated H_2_ dissociation with hydrogen spillover onto the oxide surface. The resulting hydrogen species facilitates C–O bond cleavage and oxygen-vacancy regeneration, thereby maintaining the redox cycle and active-site availability, even though the net rate of oxygen vacancy formation is expected to be low.^64,65^ Based on the proposed elementary steps in Scheme S1, hydrogen addition to adsorbed EVOH at an oxygen-vacancy site is consistent with the observed H_2_ rate dependence over Pd/MoO_3_. The H_2_ reaction order being slightly below 0.5 may reflect the contribution of a parallel pathway involving kinetically relevant dehydration on acidic support sites, followed by rapid hydrogenation over Pd sites.

The proposed elementary steps for EVOH deoxygenation over Pd/MoO_3_ are detailed in Scheme S1. The active sites on Pd/MoO_3_ include Pd metal sites, oxygen vacancies on the MoO_3_ support, and metal–support interface sites, each contributing uniquely to the catalytic process.^66,67^ The reaction begins with H_2_ dissociation on Pd sites, producing atomic hydrogen that spills over to the MoO_3_ support. This spillover facilitates the formation of oxygen vacancies on the MoO_3_ surface and at the Pd/MoO_3_ interface. EVOH adsorbs onto these vacancies, particularly at the interfacial sites, where a strong coordination with the reduced MoO_3_ surface sites near Pd facilitates selective C–O bond cleavage. Oxygen is removed via water formation, and subsequent hydrogenation of the surface-bound intermediate yields deoxygenated alkane-like polymer product. Finally, water desorbs from the catalyst surface, regenerating the active sites for subsequent reaction cycles. It is noteworthy to point out that the concentration of oxygenated side products, such as ketones, remains low across the different H_2_ partial pressures in the biphasic system. Aldehyde and ketone formation likely proceeds through a sequential partial-deoxygenation pathway. The initial step involves C–OH bond cleavage, which typically occurs at the metal–support interface. Under conditions that favor partial deoxygenation, side dehydration reactions can generate intermediates such as alkenes or cyclic ethers. These intermediates may undergo further transformation on acid sites through dehydration-mediated pathways involving double-bond migration and keto–enol tautomerism, ultimately yielding more stable aldehyde or ketone products (Figure S23).^68^

For a nonreducible support like SiO_2_, the experimental results revealed significantly lower rates, indicating weak catalytic activity. This low activity can be attributed to the inert, nonreducible nature of SiO_2_, which lacks the ability to form oxygen vacancies, which are critical for facilitating C–O bond cleavage through redox chemistry and for weakening the C–O bond at the metal–support interface.^69,70^ The observed negative rate order with respect to H_2_ flow further supports this interpretation, suggesting that increasing H_2_ partial pressure inhibits the reaction rate. This behavior may arise from the requirement to form Pd–C bonds before the rate-determining step, unlike the case for Pd/MoO_3_.^69^ To cleave the C–O bond, a prerequisite C–H bond cleavage must occur to form adequate binding to weaken the C–O bond, resulting in a negative apparent order with respect to H_2_.^71^ In contrast, for Mo-based catalysts, the affinity of the oxygen-containing group in EVOH for undercoordinated Mo sites near the metal particle promotes more rapid C–O scission. This implies that, while the active sites likely reside close to the metal–support interface, the role of the Pd is primarily to provide hydrogen in the vicinity of the metal particle, shifting the hydrogen rate dependence to a positive order. These findings reveal that Pd/SiO_2_ exhibits limitations that contrast with the enhanced activity of Pd/MoO_3_, highlighting the critical role of oxygen-vacancy formation and hydrogen spillover in maintaining catalytic activity, over Pd/MoO_3_, where continuous redox cycling and surface site availability facilitate efficient C–O bond activation and subsequent scission.

To elucidate the role of support acidity in the deoxygenation mechanism, Pd/Al_2_O_3_, an acidic catalyst support, was additionally evaluated under identical biphasic conditions. As shown in Figure S24, Pd/Al_2_O_3_ exhibited a negative reaction order with respect to H_2_ of −0.18, similar to that observed for the inert Pd/SiO_2_ benchmark. In contrast, Pd/MoO_3_ showed a positive half-order dependence on H_2_, indicating that the observed kinetic behavior cannot be attributed solely to acidity.^72,73^ Rather, the distinct kinetic response of Pd/MoO_3_ reflects Pd-assisted H_2_ dissociation, hydrogen spillover, and the generation of oxygen vacancies at the metal–support interface, with these vacancies contributing directly to the rate-determining C–O hydrogenolysis step.

Temperature-dependent initial-rate measurements were conducted under identical biphasic GVL/decalin conditions at 195, 200, and 205 °C while maintaining constant H_2_ flow and EVOH concentration, as presented in Figure 9. These measurements were performed to further clarify the mechanistic origin of the enhanced deoxygenation activity observed for Pd-promoted MoO_3_ catalysts. Among the catalysts examined, Pd5%/MoO_3_ and Pd2%/MoO_3_ exhibited substantially lower apparent activation energies of 48.9 and 54.40 kJ mol^–1^, respectively, compared with MoO_3_ (80.10 kJ mol^–1^) and Pd/SiO_2_ (91.96 kJ mol^–1^). These reduced barriers support the superior catalytic performance of the Pd/MoO_3_ materials and indicate that Pd incorporation significantly lowers the energetic requirement of the rate-determining step.

**Figure 9:**
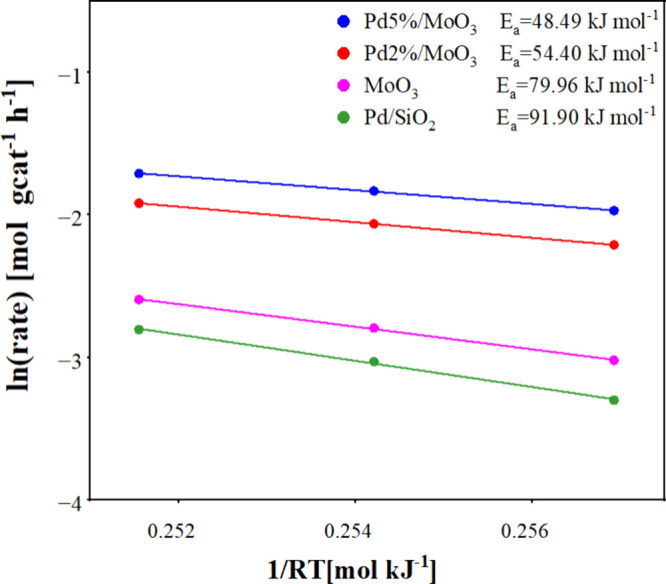
Arrhenius plots for EVOH deoxygenation over different catalysts. Reaction conditions: EVOH = 100 mg, catalyst = 10 mg, P_H_2_ = 0.33 atm, F_total = 60 mL min^–1^, *T* = 195, 200, and 205 °C.

Together with the kinetic results, including the near-zero H_2_ reaction order on unpromoted MoO_3_ and the positive half-order dependence observed on Pd/MoO_3_, these findings support a mechanism in which Pd facilitates H_2_ dissociation and promotes hydrogen spillover, thereby generating and sustaining oxygen vacancies at the metal–support interface.^74^ In contrast, the higher activation barrier of bare MoO_3_ is consistent with its limited low-temperature reducibility and weaker capacity for hydrogen activation, whereas the larger barrier measured for Pd/SiO_2_ highlights the essential role of a reducible oxide support in enabling redox cycling and stabilizing vacancy-mediated interactions with EVOH.^75^

Collectively, these activation-energy differences identify interfacial Pd/MoO_3_ sites as the dominant active centers, explain the higher oxygen-removal rates observed for these catalysts, and point to further opportunities for catalyst optimization through tuning Pd loading or support morphology while preserving selectivity during multilayer-film upcycling. To verify that the measured rates reflected intrinsic kinetics rather than transport limitations, additional control experiments were performed by varying the stirring speed and H_2_ flow rate while holding all other parameters constant. The initial rates for Pd/MoO_3_ remained unchanged across the tested stirring-speed range for Pd/MoO_3_ (Figure S25), confirming the absence of external mass-transfer limitations under the conditions employed.

## 4 Conclusion

In summary, this study demonstrates that Pd/MoO_3_ catalysts exhibit promising potential for the selective deoxygenation of oxygen-rich polymers such as EVOH, a key component in multilayer plastic films. This approach combines the deoxygenation capability of Mo-based catalysts with a biphasic solvent system to selectively remove oxygen from EVOH while improving product separation and catalyst-surface regeneration. Compared with the monophasic GVL system, the biphasic GVL/decalin system enhanced oxygen removal and enabled rapid partitioning of nonpolar PE-like products into the decalin-rich phase, thereby reducing product accumulation on the catalyst surface. When applied to commercial multilayer films containing LDPE and EVOH, the Pd5%/MoO_3_ catalyst achieved 83.5% oxygen removal after 1 h under biphasic reaction conditions. These results highlight the effectiveness of reducible metal oxide catalysts for upgrading oxygen-containing barrier layers into more compatible PE-like streams. The biphasic strategy also suggests opportunities for future process integration, including replacing the decalin phase with a PE-rich phase to reduce solvent-separation requirements in continuous processing. Overall, this work provides a catalytic pathway for improving the recyclability of multilayer plastic films and supports the broader development of sustainable plastic upcycling technologies.

## Supplementary Material



## Notes

Additional experimental details, supplementary figures, NMR spectra, catalyst characterization data, kinetic analysis, and proposed elementary steps for EVOH deoxygenation over Pd/MoO_3_ (PDF)
