# Supplementary material for: Catalytic Upcycling of Barrier Plastic Films by Selective Deoxygenation of Ethylene Vinyl Alcohol over Pd/MoO3
Source: ACS Sustain Chem Eng. 2026 Jun 1;14(26):11547–61. doi: 10.1021/acssuschemeng.5c13705 (PMC13343735; doi:10.1021/acssuschemeng.5c13705)
Supplement: Supplementary file 1 [file sc5c13705_si_001.pdf]

Supporting Information

Catalytic Upcycling of Barrier Plastic Films by Selective Deoxygenation of Ethylene Vinyl Alcohol over Pd/MoO_3

*Samira Abdolbaghi,[1] Laura A. Gomez, [1] Dai-Phat Bui,[1] Mohammad Reza Razzaghi[1], Kevin Nelson,[2] Lance L. Lobban,[1] Steven P. Crossley[1]**

[1] School of Sustainable Chemical, Biological and Materials Engineering, University of Oklahoma, Norman, Oklahoma 73019, United States

[2] Amcor, Neenah Innovation Center, Neenah, WI 54956, United States

---

Number of pages: 25
Number of figures: 25
Number of tables: 5

Table of Contents

1. EVOH ^{1}H NMR quantification and pristine EVOH spectrum S3

2. Monophasic product-separation procedure S4

3. Specific components of the multilayered film S4

4. Separation of Polar and Nonpolar Polymers S5

5. Calibration-based ^{1}H NMR determination of reacted EVOH/PE composition S5

6. Pd/MoO_3 catalyst characterization S6

7. Solventless EVOH deoxygenation by TGA S12

8. Supplementary ^{1}H NMR spectra and functional-group analyses S14

9. GVL stability under reaction conditions by ^{1}H NMR and GC–MS S16

10. Quantitative analysis of multilayer-film products S19

11. Comparative catalytic performance and kinetic analysis S20

12. Proposed elementary steps and kinetic derivation for EVOH deoxygenation over Pd/MoO_3 S23

13. References S25

1. EVOH ¹H NMR quantification and pristine EVOH spectrum

The quantity of each species is determined by integrating the ^{1}H NMR peaks corresponding to each functional group (e.g., R–CH(OH)-, R–CH_2-, and R–C(OH)-) and the number of hydrogen atoms associated with each group. The quantity of residual oxygen-containing species is estimated using signals corresponding to RC-(OH) and R-CH(OH)- groups. For each R-C(OH)- group, the stoichiometric count of protons bound to carbon in the R-CH(OH)- functional group is subtracted from the total R-C(OH)- peak in that region. This subtraction isolated the remaining protons, which are attributed to aldehyde or ketone groups. The relative abundance of each species in EVOH is calculated by normalizing the integrated ^{1}H NMR data for each species to the total signal.

Table S1. ^{1}H NMR calculations for pristine EVOH based on functional group distribution.

	R-CH_3	R-C**H_2**-	R-C(O**H**)-	R-C**H**(OH)-	Ether & Ketone
Area	432	61098	15500	15650	15
Number of Protons	3	2	1	1	4
Normalized area (Area/Number of Protons)	144	30549	15500	15650	
Total Area			46193=144+30549+15500		
% (Normalized area /Total Area)	0.31%	66.1%	33.5%	NA	0.0

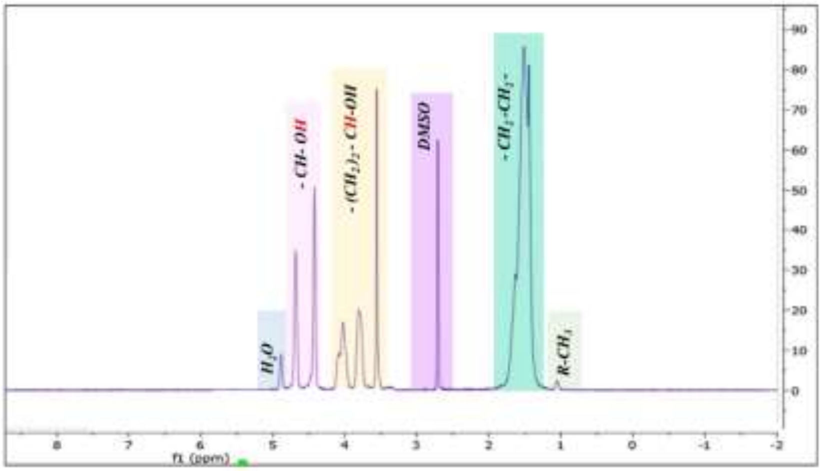

Figure S1. ^{1}H NMR spectrum of pristine EVOH in DMSO-d_6

2. Monophasic product-separation procedure

Upon completion of the experiment, the reactor was cooled down to ambient temperature using an ice bath. The solid fraction was initially separated from the solvent via centrifugation at 11,000 rpm for 15 min. The isolated solid was dissolved in dimethyl sulfoxide (DMSO) and subjected to a second centrifugation at 11,000 rpm for 30 min to separate the catalyst from the remaining components. The resulting supernatant was introduced into water to precipitate the polymer product. To ensure the purity of the polymer, the precipitate underwent three cycles of centrifugation at 11,000 rpm for 5 min and was then washed with water. The final polymer product was subsequently dried under vacuum at 80 °C overnight. This meticulous purification protocol was designed to eliminate all traces of GVL, ensuring the integrity and purity of the polymer product for subsequent analytical characterization.

3. Specific components of the multilayered film

The multilayered profile consists of the following layers: LDPE (low-density polyethylene), LDPE (low-density polyethylene), LLDPE-g-MAh (linear low-density polyethylene grafted with maleic anhydride), EVOH (ethylene vinyl alcohol copolymer), LLDPE-g-MAh (linear low-density polyethylene grafted with maleic anhydride), LDPE (low-density polyethylene), and LDPE (low-density polyethylene). The composition, density, and relative layer fraction of each polymer in the multilayer structure are summarized in Table **S2**.

Table S2. Components of the commercial multilayered film

	LDPE	LDPE	LLDPE-g-MAh	EVOH	LLDPE-g-MAh	LDPE	LDPE	Total
Density (g/cm^3)	0.923	0.923	0.918	1.19	0.918	0.923	0.923	0.965
Layer content by mass (%)	15	15	10	20	10	15	15	100
Layer content by volume (%)	15.7	15.7	10.5	16.2	10.5	15.7	15.7	100
Relative layer thickness (%)	15.7	15.7	10.5	16.2	10.5	15.7	15.7	100

4. Separation of Polar and Nonpolar Polymers

The procedure for isolation of the reaction products from the film involves several steps. First, the total mass of the reaction products was precisely recorded. The mixture was fractionated into polar and nonpolar polymers. Nonpolar polymers are extracted by dissolving the reaction products in decalin at 40 °C for 8 hours. To purify the nonpolar species, the solution underwent two acetone washing cycles, followed by centrifugation at 11000 rpm for 10 minutes. The remaining solid, consisting of polar polymers and the catalyst, was dissolved in DMSO at 60 °C for 8 h. The solution was subjected to two sequential acetone washes and centrifuged at 11,000 rpm for 10 minutes. Both polymer fractions are thoroughly dried, and their masses were recorded. For ^{1}H NMR analysis, the polar product was dissolved in DMSO-d_6 at 80 °C for 5 hours, while the non-polar product was dissolved in $CDCl_3$ at 40 °C for 5 h.

5. Calibration-based ^{1}H NMR determination of reacted EVOH/PE composition

The composition of the reacted EVOH/PE mixtures was determined using a calibration-based ^{1}H NMR method. Standard mixtures of pristine EVOH and PE with known mass compositions were prepared and analyzed under identical ^{1}H NMR conditions to construct calibration curves. For each standard mixture, the response ratio R was calculated from the integrated NMR peak areas according to Equation S1, where I(OH) corresponds to the integral area of the EVOH hydroxyl signal, and I(CH_2) corresponds to the integral area of the methylene signal of the mixture. The calibration curve was generated by plotting the measured R values against the known EVOH mass fractions of the standard mixtures, as shown in Figure **S2**. Linear regression of these data provided the fitting parameters a and b, which define the relationship given in Equation S2. The EVOH content of each reacted sample was then obtained by substituting its measured R value into Equation S2. The calibrated compositions of the recovered polymer mixtures were subsequently used for mass-balance analysis and for the determination of EVOH conversion and PE content.

$$R = \frac{I_{OH}}{I_{OH}+I_{CH_2}} \qquad \text{Eq. S1}$$

$$\text{EVOH}(wt\,\%) = 361.61R + 2.33 \qquad \text{Eq. S2}$$

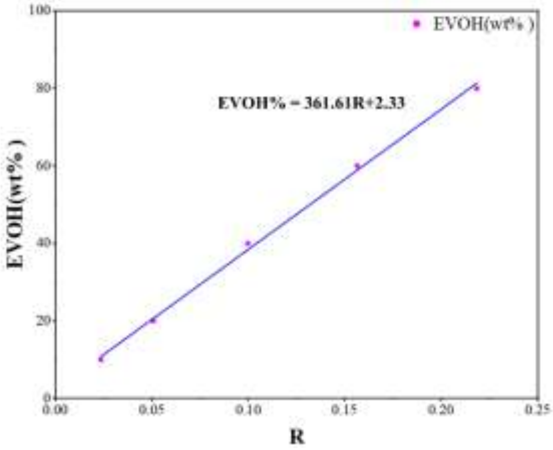

Figure S2. Calibration curve showing the response ratio R as a function of the EVOH mass fraction in pristine EVOH/PE standard mixtures

6. Pd/MoO_3 catalyst characterization

Different characterization techniques were used to evaluate oxygen-vacancy concentration, catalyst reducibility, surface area, morphology, and elemental distribution of the Pd/MoO_3 catalysts. O_2 chemisorption was used as a probe molecule to quantify the number of oxygen vacancies formed under H_2 reduction conditions. Several studies indicate that surface defects on MoO_3 favor O_2 dissociation, which leads to the titration of two oxygen vacancies, even at low temperatures. Table S**3** indicates the total number of oxygen vacancies at the surface formed for both samples, Pd2%/MoO_3 and Pd5%/MoO_3.

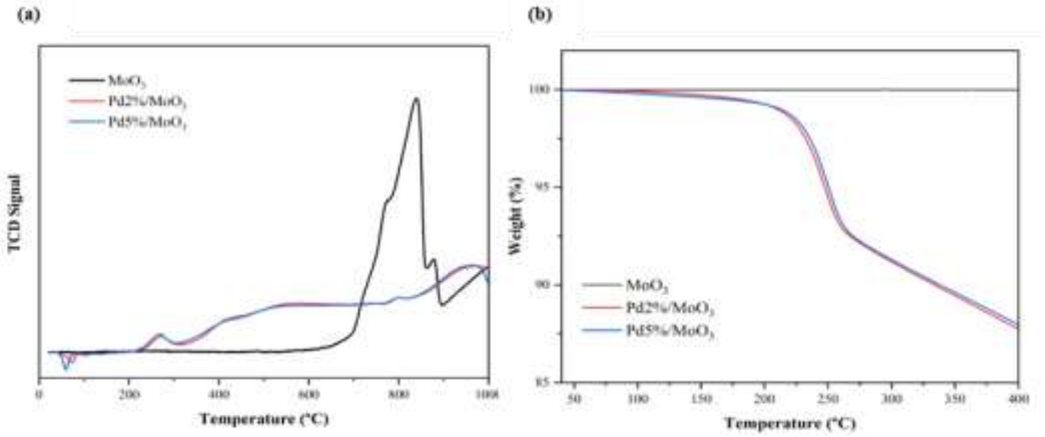

Figure S3. (a) TPR and **(b)** TPR-TGA profiles of MoO_3, Pd2%/MoO_3, and Pd5%/MoO_3.

Table S3. Summary of O_2 chemisorption measurements used to estimate oxygen-vacancy formation for Pd2%/MoO_3 and Pd5%/MoO_3 catalysts.

**Measured Quantity** (μmol g^{-1})	**Pd2%/MoO_3**	**Pd5%/MoO_3**
Number of O_2 adsorbed	1618.3	1576.7
Number of H_2O produced	303.9	300.4
O_2 chemisorbed over Pd/SiO_2	62.3	155.8
Total amount of O_2 chemisorbed over MoO_{3-x}	1252.14	1120.4

Additional HAADF-STEM images of Pd2%/MoO_3 and Pd5%/MoO_3 are provided in Figures **S4** and **S5**, respectively, to further show the Pd particle distribution on the MoO_3 support.

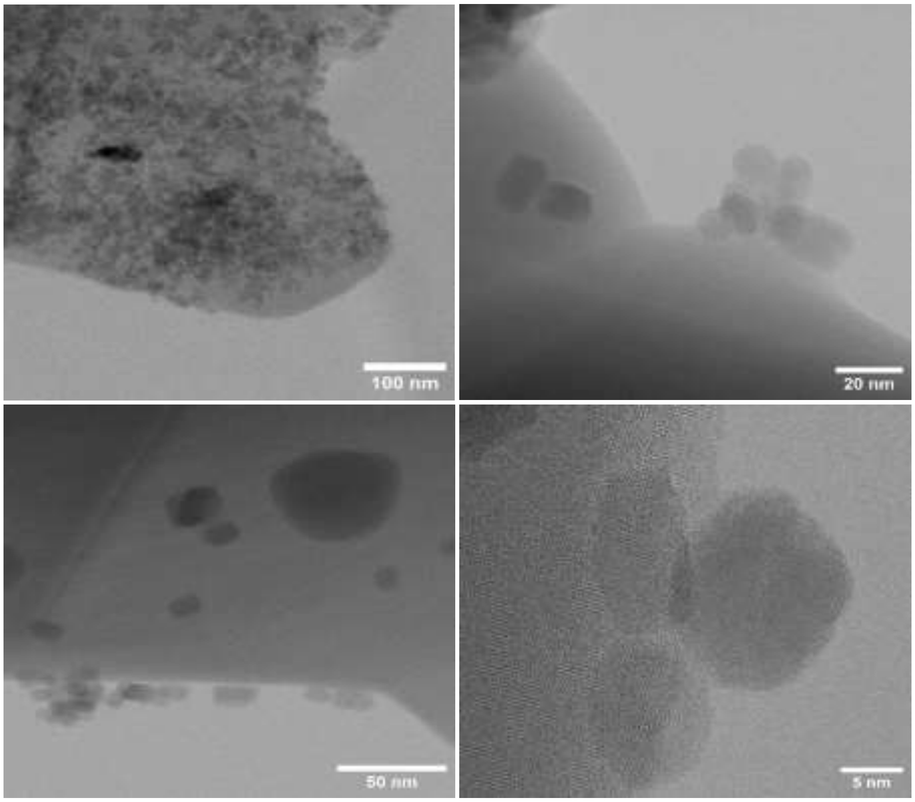

Figure S4. Representative STEM images of Pd2%/MoO_3.

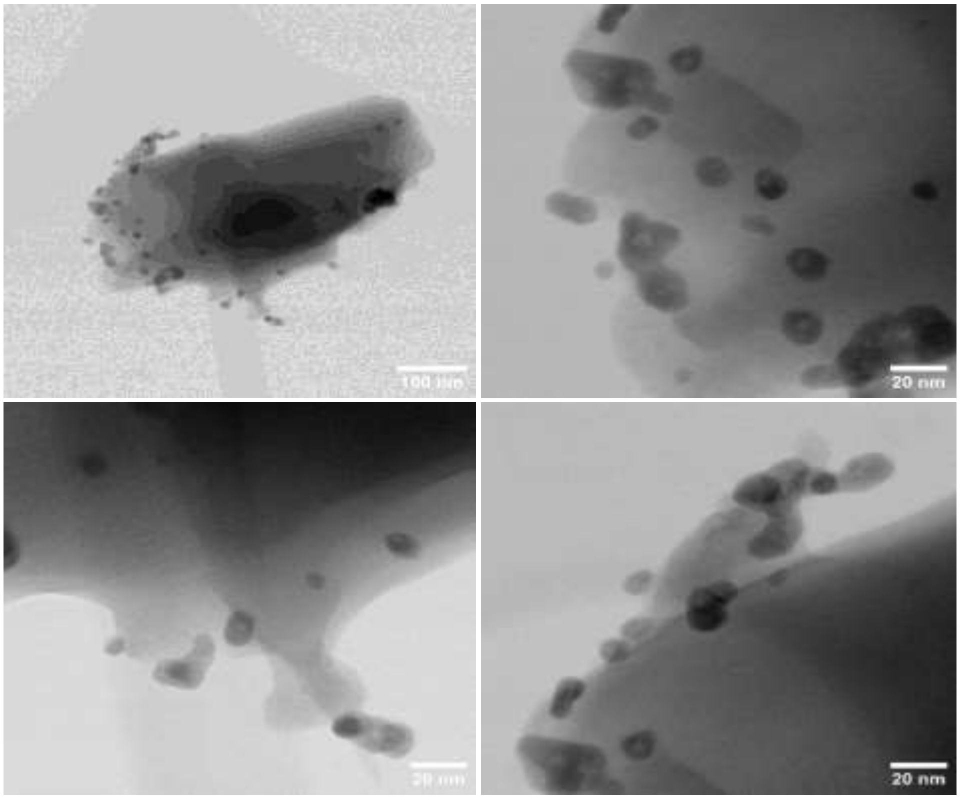

Figure S5. Representative STEM images of Pd5%/MoO_3.

The BET surface area data for the reduced catalysts are presented in Table **S4**. The results indicate that Pd incorporation increases the accessible surface area of the reduced MoO_3 catalyst. This trend suggests that palladium affects not only hydrogen activation and surface hydrogen availability but also the extent of catalyst surface accessible to reactants, which may contribute to the improved catalytic performance.

Table S4. BET specific surface area of reduced MoO_3, reduced Pd2%/MoO_3 and Pd5%/MoO_3 catalysts

**Catalyst**	**Surface area (m^2 g^{-1})**	**Pore volume (cm^3 g^{-1})**	**Micropore volume (cm^3 g^{-1})**
MoO_3	2.01	0.00048	0.00001
Pd2%/MoO_3	3.80	0.0031	0.00051
Pd5%/MoO_3	6.45	0.0121	0.00091

The SEM–EDS spectra and corresponding elemental mapping results for the Pd2%/MoO_3 and Pd5%/MoO_3 catalysts are shown in Figures **S6** and **S7**, respectively. The maps confirm the presence of Mo, O, and Pd and indicate a relatively uniform distribution of Pd over the MoO_3 support.

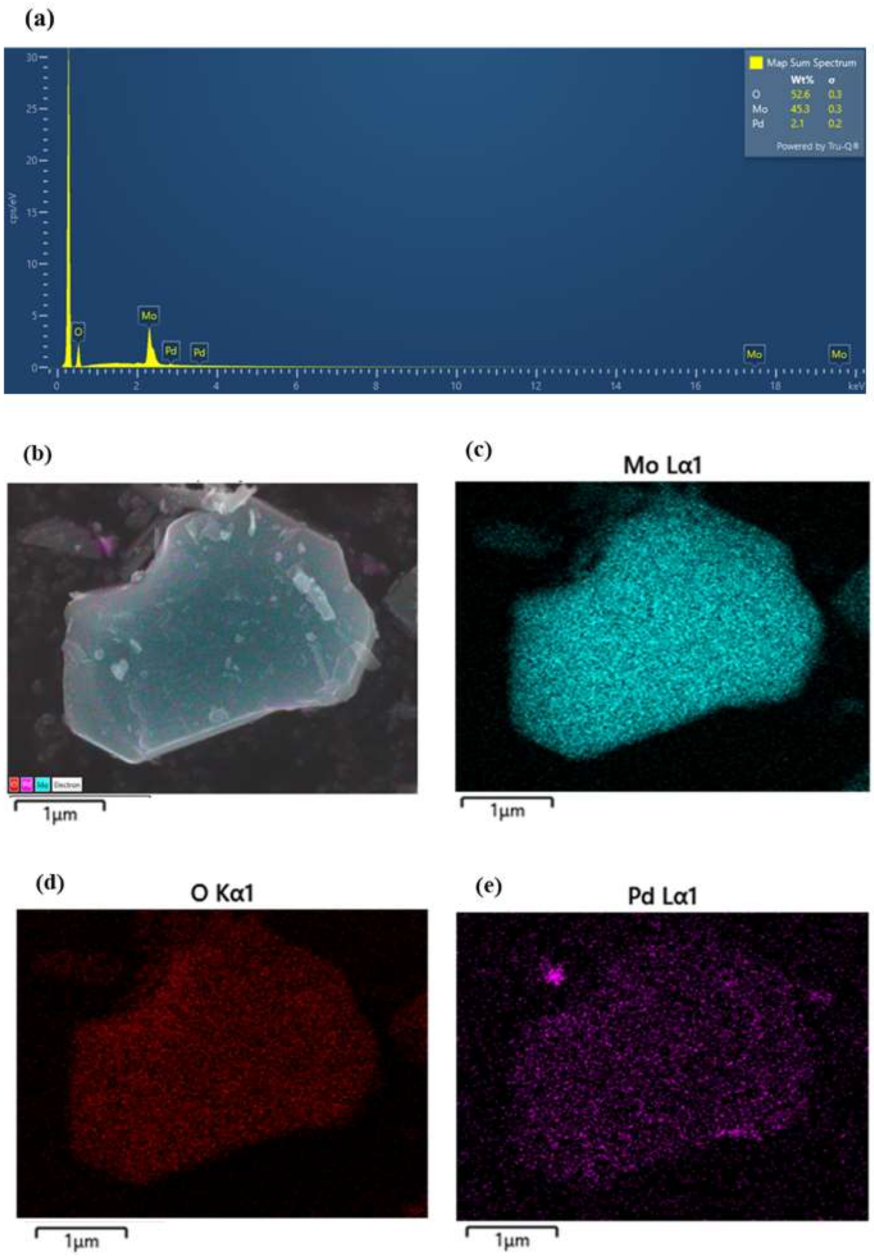

Figure S6. (a) EDS spectrum, (b) SEM image, and corresponding SEM–EDS elemental maps of the Pd2%/MoO_3 catalyst: (c) Mo, (d) O, and (e) Pd.

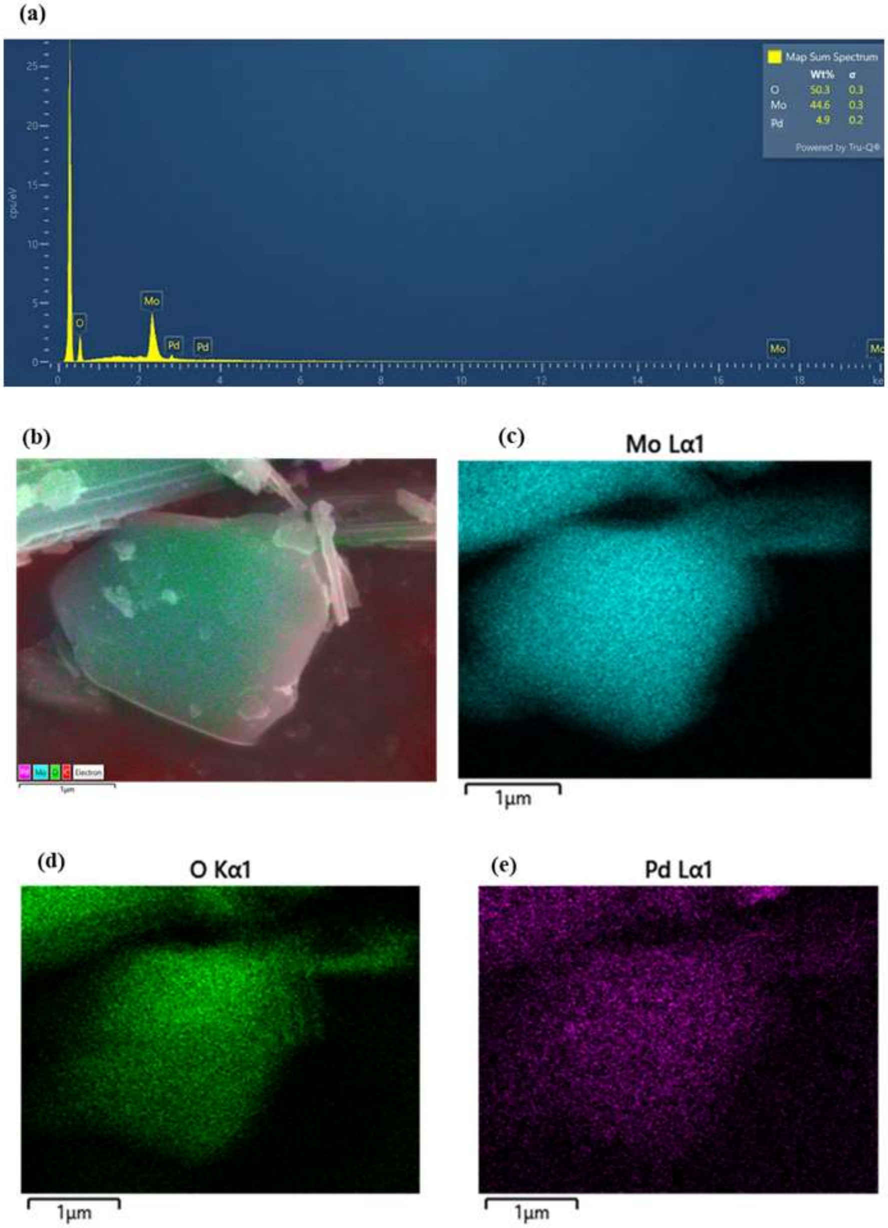

Figure S7. (a) EDS spectrum, (b) SEM image and corresponding SEM–EDS elemental maps of the Pd5%/MoO_3 catalyst: (c) Mo, (d) O, and (e) Pd.

The XRD results of fresh MoO_3, calcined MoO_3 (600 °C), fresh Pd/MoO_3, and spent Pd/MoO_3 are shown in Figure **S8**. The diffraction patterns remain highly similar and are clearly distinct from that of MoO_2, indicating that no detectable bulk phase transformation occurred under the calcination or reaction conditions.

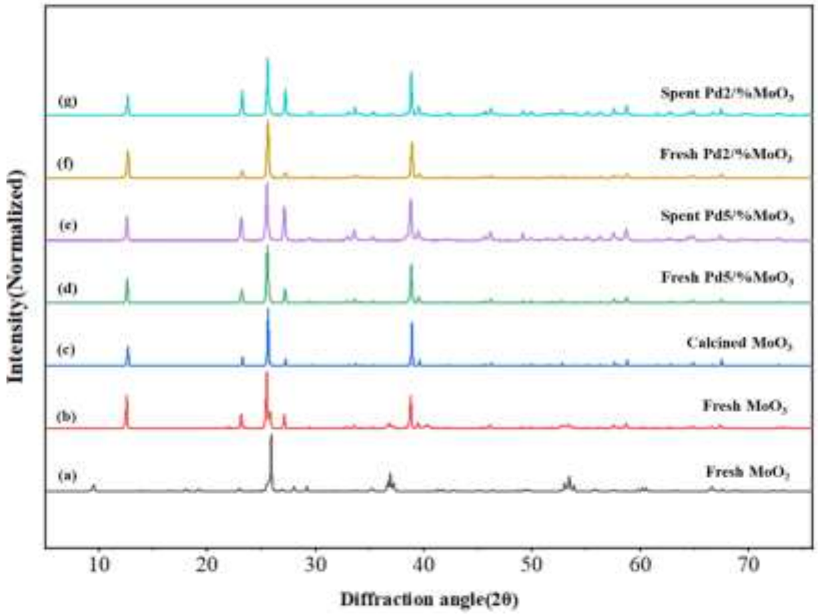

Figure S8. XRD patterns of fresh MoO_3, calcined MoO_3 (600 °C), fresh Pd2%/MoO_3, fresh Pd5%/MoO_3, spent Pd2%/MoO_3, spent Pd5%/MoO_3, and MoO_2 as a reference sample.

The TGA-TPO profiles of the spent Pd2%/MoO_3 and Pd5%/MoO_3 catalysts are shown in Figure **S9**. Only minor mass losses of approximately 0.4 and 0.5 wt %, respectively, were observed, indicating a low extent of coke or other carbonaceous deposits formed during the reaction.

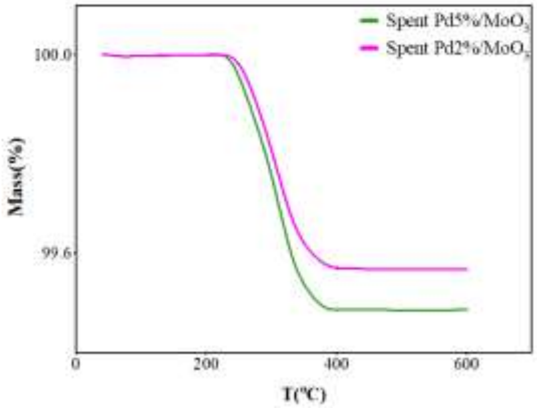

Figure S9. TGA–TPO profiles of spent Pd2%/MoO_3 and Pd5%/MoO_3 catalysts.

7. Solventless EVOH deoxygenation by TGA

The blank TGA experiment of Pd/MoO_3 under H_2 without EVOH showed negligible mass loss under the reaction conditions (Figure **S10**), indicating that catalyst reduction makes only a minor contribution to the observed mass loss. Accordingly, the additional mass loss in the EVOH-containing samples is attributed mainly to polymer deoxygenation. The TGA results further showed that the extent of EVOH deoxygenation increased with catalyst loading for both Pd2%/MoO_3 and Pd5%/MoO_3 (Figure **2** in the main text and Figure **S10**). In addition to the higher overall hydroxyl-group removal, the deoxygenation rate normalized per Pd atom also increased with catalyst loading. This behavior suggests that catalyst loading influences not only the number of available active sites but also the ability to maintain selective deoxygenation under melt-phase conditions. At lower loadings, the limited density of Pd/MoO_3 interfacial sites may promote the buildup of partially deoxygenated intermediates near the catalyst surface, thereby decreasing the effective turnover rate. By contrast, higher loadings likely provide a greater density of sites for hydrogen dissociation, spillover, and oxygen-vacancy regeneration, enabling more sustained C–O bond cleavage and hydrogenation.

To further elucidate the reaction pathway under solventless conditions, the products recovered from the TGA crucibles were analyzed by ^{1}H NMR, and the corresponding functional group distributions are presented in Figure **S11**. In addition to the intended reduction in hydroxyl-containing groups, small quantities of oxygenated byproducts, specifically ketone and ether-type species, were detected. Their presence suggests that, under melt-phase conditions, partial deoxygenation of EVOH may generate unsaturated or incompletely hydrogenated intermediates, which subsequently undergo secondary transformations before complete conversion to polyethylene-like chains. One plausible pathway involves keto–enol tautomerization of intermediate unsaturated alcohol species, giving rise to ketone-containing structures. Ether formation may arise from condensation-type reactions between partially deoxygenated chains. Collectively, these observations indicate that, while Pd/MoO_3 is active for solventless EVOH deoxygenation, the absence of a solvent may promote local accumulation of intermediates and facilitate secondary reactions that compete with complete hydrogenolysis and hydrogenation.

The reduced efficiency of the solventless TGA system compared with liquid-phase reaction systems is likely attributable to transport limitations and secondary reaction effects in the molten-polymer environment. In the absence of solvent, the catalyst operates within a highly viscous medium, which can impede H_2 diffusion and restrict contact between EVOH chains and catalytically active sites. Under these conditions, partially deoxygenated intermediates may persist near the catalyst surface, increasing the likelihood of secondary condensation or coupling reactions that decrease catalyst effectiveness. This behavior may explain why the enhancement in deoxygenation with increasing catalyst loading exceeds

what would be expected from Pd content alone. Consequently, the solvent-based systems discussed in the main text are important not only for achieving higher overall conversion but also for alleviating melt-phase transport constraints and suppressing the accumulation of reactive intermediates.

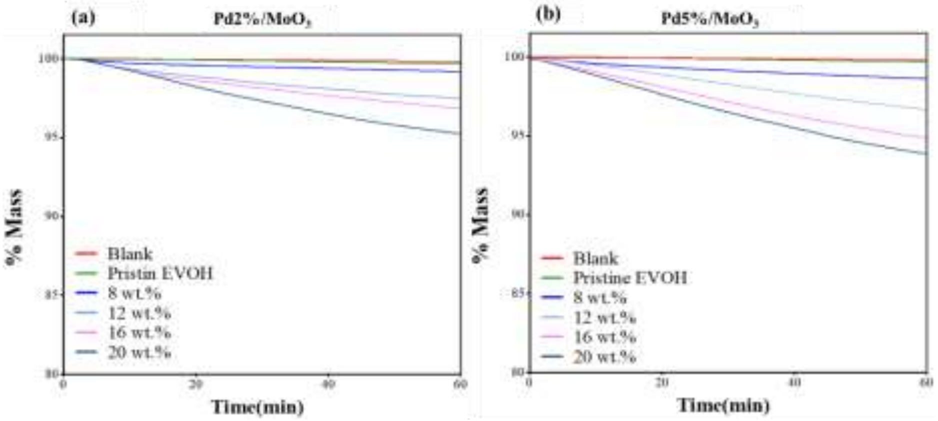

Figure S10. TGA mass-loss profiles for the solventless deoxygenation of EVOH under H_2 over Pd2%/MoO_3 and Pd5%/MoO_3, including pristine EVOH and blank catalyst controls without EVOH. Reaction conditions: EVOH = 20 mg, catalyst loading = 8–20 wt % relative to EVOH, H_2 = 40 mL min^{-1}, and T = 200 °C.

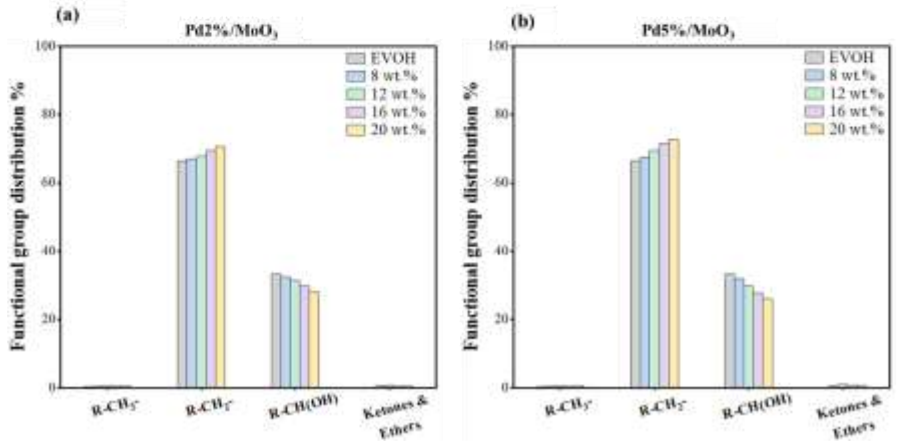

Figure S11. Functional-group distribution of products obtained after solventless EVOH deoxygenation by TGA under H_2 over (a) Pd2%/MoO_3 and (b) Pd5%/MoO_3. Reaction conditions: EVOH = 20 mg, catalyst loading = 8–20 wt % relative to EVOH, H_2 = 40 mL min^{-1}, and T = 200 °C.

8. Supplementary ^{1}H NMR spectra and functional-group analyses

Figure **S12** presents ^{1}H NMR spectra of the reaction products obtained from EVOH deoxygenation in the monophasic reaction system over Pd2%/MoO_3 and Pd5%/MoO_3 in DMSO-d_6. The decrease in the R–CH(OH)– signal and the increase in the R–CH_2– signal relative to pristine EVOH indicate progressive C–O bond cleavage and hydrogenation during the reaction.

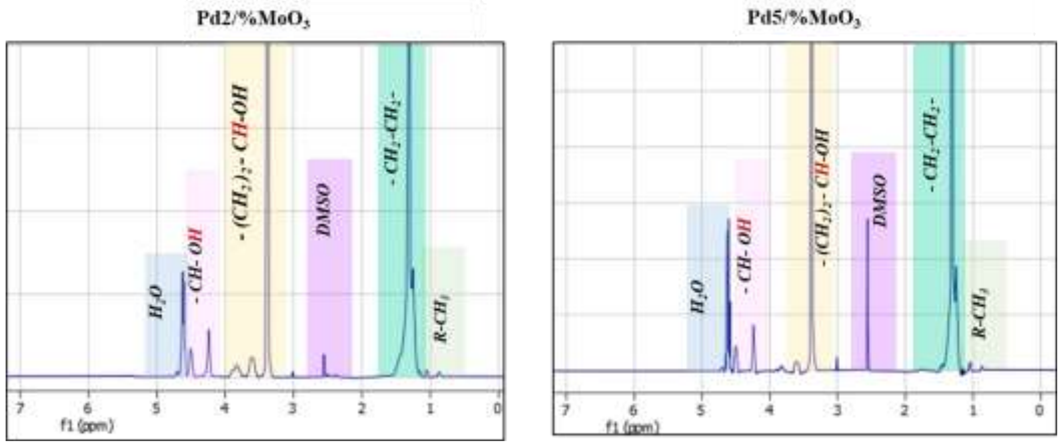

Figure S12. ^{1}H NMR spectra of EVOH reaction products from the monophasic catalytic reaction over (a) Pd2%/MoO_3 and (b) Pd5%/MoO_3. Reaction conditions: EVOH = 100 mg, catalyst = 10 mg, F_H_2 = 60 mL min^{-1}, T = 200 °C, and reaction time = 4 h.

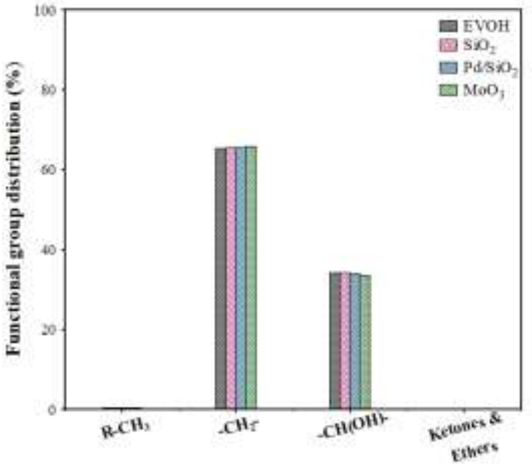

Figure S13. Functional-group distribution (%) of EVOH reaction products over SiO_2, Pd/SiO_2, and MoO_3. Reaction conditions: EVOH = 100 mg, catalyst = 10 mg, F_H_2 = 60 mL min^{-1}, T = 200 °C, and GVL = 10 mL.

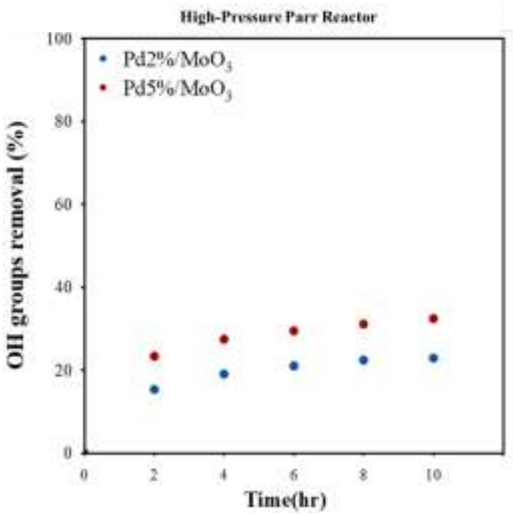

Figure S14. Hydroxyl-group removal (%) from EVOH as a function of time in the monophasic system in a Parr reactor under high H_2 pressure. Reaction conditions: EVOH = 100 mg, catalyst = 10 mg, P_H_2 = 400 psi, T = 200 °C, and GVL = 10 mL.

9. GVL stability under reaction conditions by ^{1}H NMR and GC–MS

The stability of GVL under the reaction conditions was verified by blank experiments containing GVL, catalyst, and H_2 in both biphasic and monophasic systems. As shown in Figure **S15a–c**, the ^{1}H NMR spectra of fresh and recovered GVL were essentially identical, with no visible changes in the characteristic solvent peaks. In addition, no measurable liquid mass loss was observed after reaction. GC–MS analysis of fresh and recovered GVL from the monophasic blank experiment (Figure **S16 a,b**) likewise showed similar chromatograms without new detectable peaks. Although GVL can, in principle, undergo ring opening followed by hydrogenolysis or hydrogenation under catalytic hydrogenation conditions, no evidence of such transformations was observed here. These results confirm that GVL is stable under the deoxygenation conditions employed in this study.

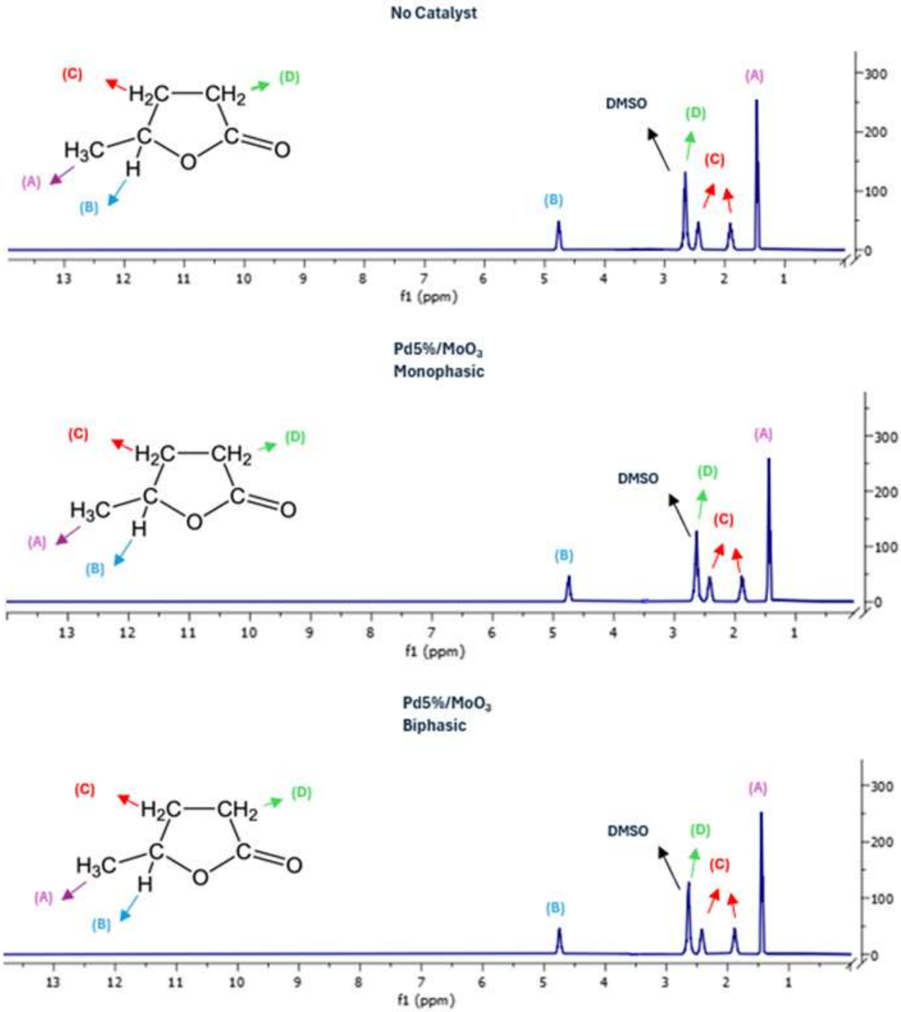

Figure S15. ^{1}H NMR spectra of (a) fresh GVL, (b) recovered GVL after the monophasic blank reaction, and (c) recovered GVL after the biphasic blank reaction, showing no detectable solvent transformation under the reaction conditions. Reaction conditions: catalyst = 10 mg, H_2 = 60 mL min^{-1}, T = 200 °C, and GVL = 10 mL; DMSO-d_6 was used as the NMR solvent.

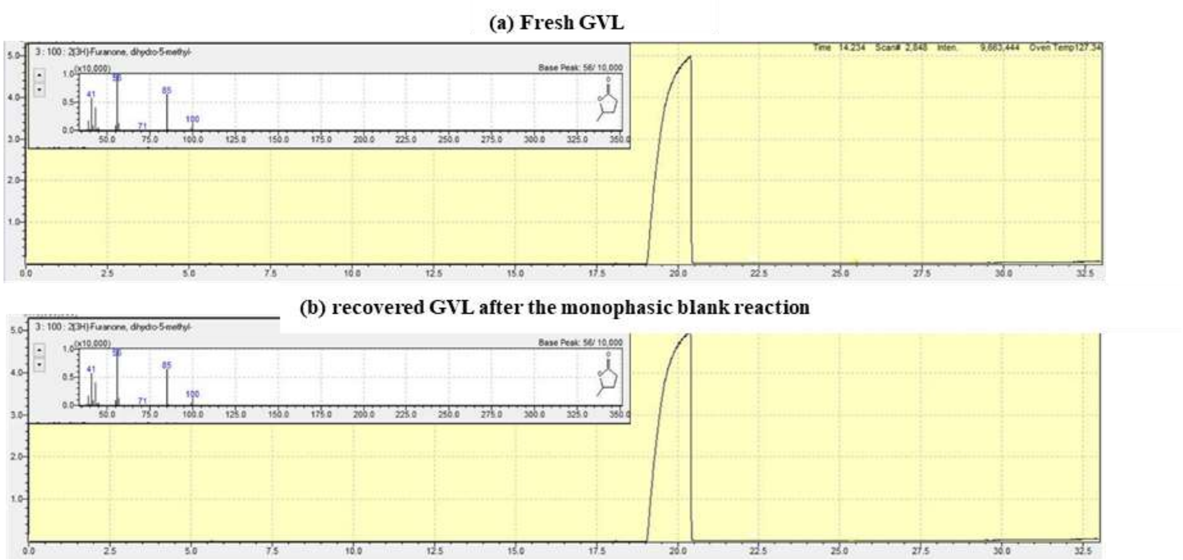

Figure S16. GC–MS chromatograms of (a) fresh GVL and (b) recovered GVL after the monophasic blank reaction (GVL + catalyst + H_2), confirming the absence of detectable solvent-decomposition products. Reaction conditions: H_2 = 60 mL min^{-1}, T = 200 °C, and GVL = 10 mL.

10. Quantitative analysis of multilayer-film products

Table S5. Quantitative ^{1}H NMR analysis of the reaction products obtained from multilayer-film deoxygenation over Pd2%/MoO_3 and Pd5%/MoO_3, including the calculated EVOH content, PE content, oxygen removal, and degree of deoxygenation.

	I(OH)	I(CH_2)	R	EVOH	PE	Oxygen removed	Deoxygenation
	Normalized area	Normalized area		%	%	%	%
**Pd2%/MoO_3**	56.77	4490	0.012	6.87	89.8	3.30	56.8
**Pd5%/MoO_3**	37.71	2085	0.017	8.75	88.4	2.82	65.29

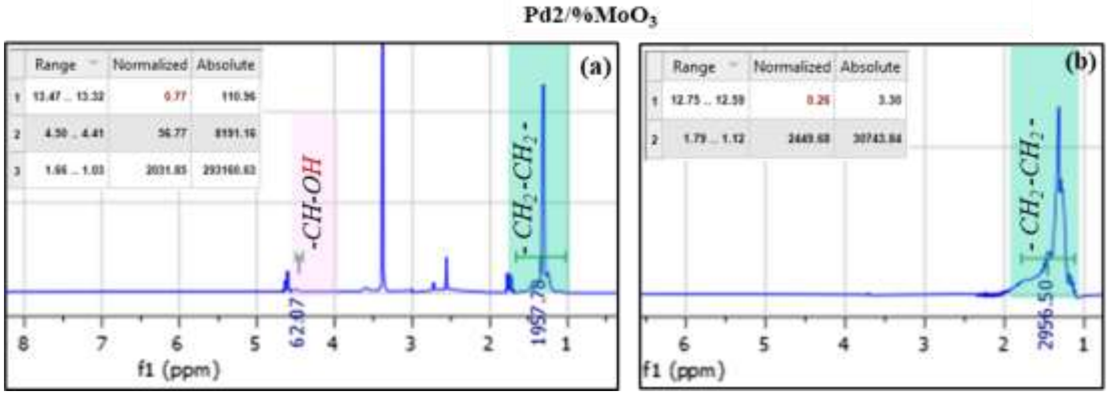

Figure S17. ^{1}H NMR spectra of the film components before and after reaction over Pd2%/MoO_3: nonpolar components dissolved in $CDCl_3$ and polar components dissolved in DMSO-d_6. (a) Before reaction and (b) after reaction. Reaction conditions: film = 100 mg, catalyst = 10 mg, H_2 = 60 mL min^{-1}, T = 200 °C, reaction time = 1 h, solvent system = 5 mL GVL and 5 mL decalin.

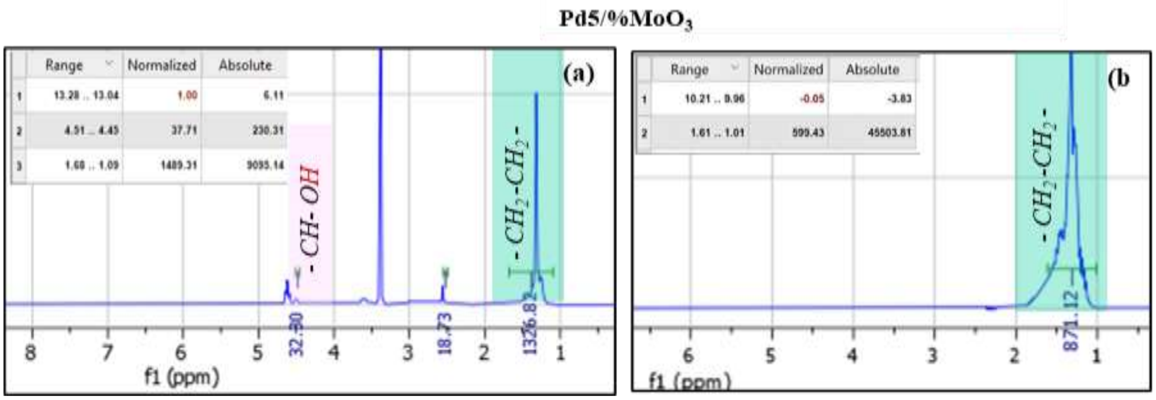

Figure S18. ^{1}H NMR spectra of the film components before and after reaction over Pd5%/MoO_3: nonpolar components dissolved in $CDCl_3$ and polar components dissolved in DMSO-d_6. (a) Before reaction and (b) after reaction. Reaction conditions: film = 100 mg, catalyst = 10 mg, H_2 = 60 mL min^{-1}, T = 200 °C, reaction time = 1 h, solvent system = 5 mL GVL and 5 mL decalin.

11. Comparative catalytic performance and kinetic analysis

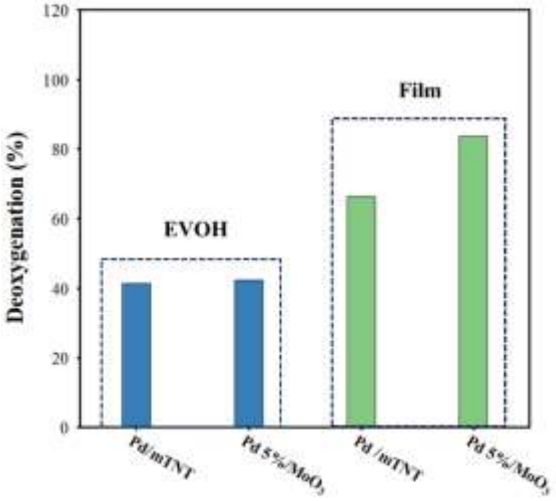

Figure S19. Oxygen removal (%) from EVOH and a commercial multilayer film over Pd5%/MoO_3 compared with Pd/m/TNT in a biphasic system. Reaction conditions: film = 100 mg, EVOH = 100 mg, catalyst = 10 mg, H_2 = 60 mL min^{-1}, T = 200 °C, reaction time = 30 min for film and 1 h for EVOH, solvent system = 5 mL GVL and 5 mL decalin. Data for Pd/m/TNT were reproduced from Bui et al (Ref. 1). [1]

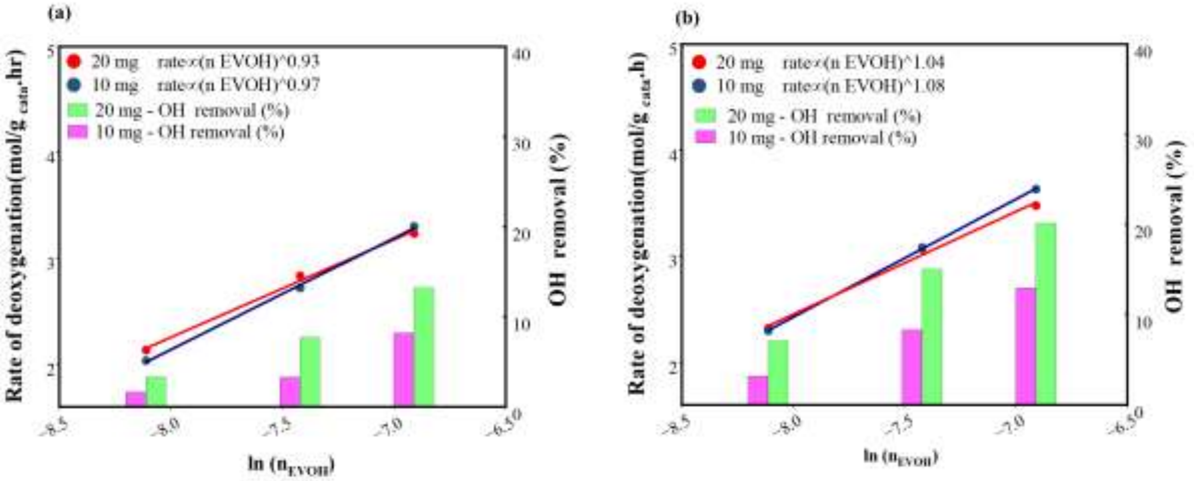

Figure S20. Reaction order with respect to EVOH over Pd/MoO_3 at different catalyst loadings: (a) Pd2%/MoO_3 and (b) Pd5%/MoO_3. Reaction conditions: catalyst = 10 or 20 mg, EVOH = 30, 60, or 100 mg, P_H₂ = 1 atm, F_total = 60 mL min^{-1}, and T = 200 °C.

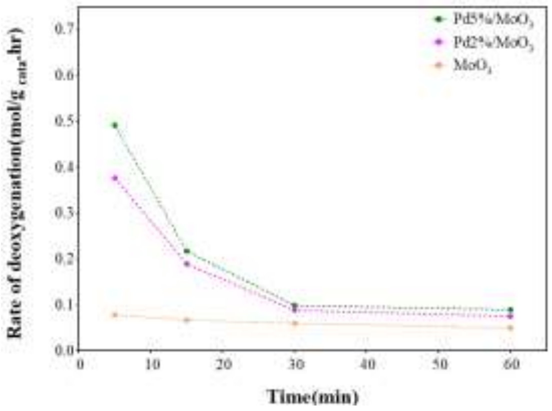

Figure S21. Deoxygenation rate as a function of reaction time for the tested catalysts. Reaction conditions: catalyst = 10 mg, EVOH = 60 mg, P_H_2 = 1 atm, F_total = 60 mL min^{-1}, and T = 200 °C.

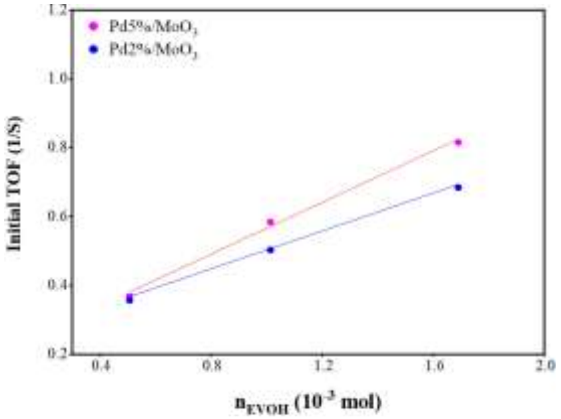

Figure S22. Turnover frequency as a function of EVOH loading. Reaction conditions: catalyst = 10 mg, EVOH = 30, 60, or 100 mg, P_H_2 = 0.6 atm, F_total = 60 mL min^{-1}, and T = 200 °C.

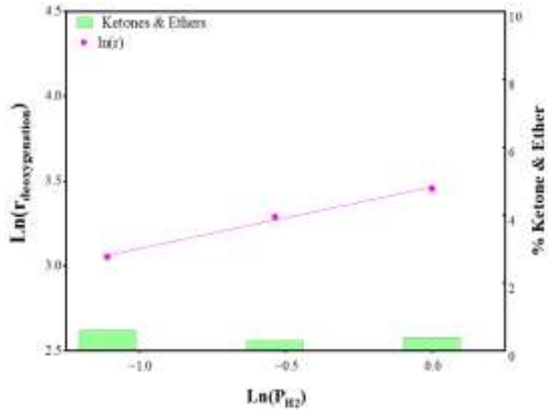

Figure S23. Effect of H_2 partial pressure on deoxygenation rate and ether/ketone formation over Pd5%/MoO_3. Reaction conditions: EVOH = 100 mg, catalyst = 10 mg, P_H_2 = 0.3, 0.6, or 1 atm, F_total = 60 mL min^{-1}, and T = 200 °C.

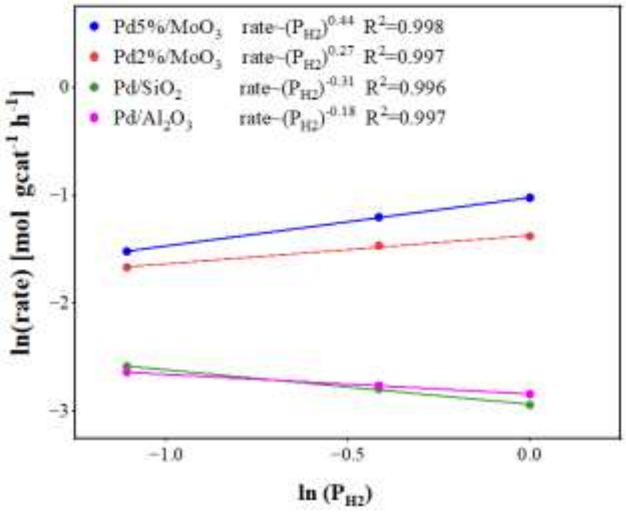

Figure S24. Comparison of H_2 reaction orders for EVOH deoxygenation over Pd/MoO_3, Pd/SiO_2, and Pd/Al_2O_3 catalysts. Reaction conditions: EVOH = 100 mg, catalyst = 10 mg, P_H_2 = 0.3, 0.6, or 1 atm, F_total = 60 mL min^{-1}, and T = 200 °C.

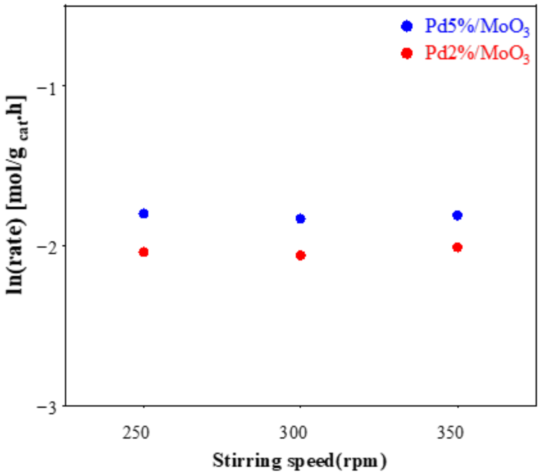

Figure S25. Effect of stirring speed on the initial rate of EVOH deoxygenation over MoO_3 and Pd/MoO_3 catalysts in the biphasic GVL/decalin system. Reaction conditions: EVOH = 100 mg, catalyst = 10 mg, P_H_2 = 0.3 atm, F_total = 60 mL min^{-1}, T = 200 °C, and stirring speed = 250, 300, or 350 rpm.

12. Proposed elementary steps and kinetic derivation for EVOH deoxygenation over Pd/MoO_3

The proposed reaction pathway for catalytic EVOH deoxygenation over Pd/MoO_3 proceeds through a series of elementary steps, as shown in Scheme **S1**. Initially, molecular hydrogen adsorbs and dissociates on Pd metal sites (*), generating atomic hydrogen that can spill over to the MoO_3 support. This spillover hydrogen promotes the formation and regeneration of oxygen-vacancy sites (∅), which are proposed to participate in C–OH bond activation, particularly at the Pd/MoO_3 interface. EVOH then adsorbs at Pd/MoO_3 interfacial sites through coordination of the hydroxyl group with reduced MoO_3 or oxygen-vacancy sites, weakening the C–OH bond. Subsequent C–OH bond cleavage and water formation generate a partially deoxygenated surface intermediate, which is then rapidly hydrogenated by spillover hydrogen to form PE-like $–CH_2–CH_2–$ units. Finally, the PE-like product desorbs from the catalyst surface, regenerating the active sites for the next catalytic cycle. The cooperative roles of Pd for H_2 dissociation and MoO_3 oxygen-vacancy sites for C–OH activation provide a mechanistic basis for the enhanced deoxygenation activity of Pd/MoO_3.

[1] $$H_2 + 2[*] \xleftrightarrow[K_1]{} 2H^*$$

[2] $$H^* + ⊛ \xleftrightarrow[K_2]{} H^{⊛} + [*]$$

[3] $$H^{⊛} + H^* \xleftrightarrow[K_3]{} H_2O^{\varnothing} + [*]$$

[4] $$[H_2O^{\varnothing}] \xleftrightarrow[K_4]{} H_2O + [\varnothing]$$

[5] $$[-CH_2CH(OH)-] + [\varnothing] \xleftrightarrow[K_5]{} [-CH_2CH(OH)-]^{\varnothing}$$

[6] $$[-CH_2CH(OH)-]^{\varnothing} + H^* \xleftrightarrow[K_6]{} [-CH_2CH-]^* + H_2O^{\varnothing}$$

[7] $$[-CH_2CH-]^* + H^* \xleftrightarrow[K_7]{} [-CH_2CH_2-]^* + [*]$$

[8] $$[-CH_2CH_2-]^* \xleftrightarrow[K_8]{} [-CH_2CH_2-] + [*]$$

Scheme S1. Proposed elementary steps for EVOH deoxygenation over Pd/MoO_3. Here, (*) denotes Pd metal sites, (∅) represents oxygen-vacancy sites on MoO_3, (→) indicates the kinetically relevant step, and (↔) indicates a quasi-equilibrated step. Descriptions of the individual elementary steps are provided below.

Step 1. H_2 adsorption and dissociation on Pd sites

Step 2. Spillover hydrogen migration from Pd to MoO_3

Step 3. Water formation at an oxygen-vacancy/interfacial site

Step 4. Oxygen-vacancy regeneration

Step 5. EVOH adsorption through the hydroxyl group

Step 6. First hydrogen addition to the surface intermediate

Step 7. Second hydrogen addition to form the PE-like unit

Step 8. Desorption of the PE-like product

Adsorption and equilibrium constants were derived assuming quasi-equilibrated adsorption and hydrogen-addition steps, while the steady-state approximation was applied to surface intermediates to obtain rate expressions for the proposed surface-reaction steps. Different possible rate-limiting steps were considered to identify the rate expression most consistent with the experimental kinetic data.

I. First hydrogen addition as the rate-limiting step (Step 6)

$$[-CH_2CH\,(OH)]^{\varnothing} + H^* \xrightarrow[K_6]{} [-CH_2CH-]^* + H_2O^{\varnothing}$$

$$r_6 = k_6\,[-CH_2CH\,(OH)]^{\varnothing}\,[H^*] \qquad \text{Eq. S3}$$

Using the adsorption constants for Steps 1 and 5, the surface coverages of the adsorbed hydrogen $[H^*]$ and EVOH-derived intermediate $[-CH_2CH\,(OH)]^{\varnothing}$ were expressed in terms of measurable reactant concentrations and substituted into the rate expression for Eq. S3.

$$K_5 = \frac{[-CH_2CH\,(OH)-]^{\varnothing}}{[-CH_2CH\,(OH)-]\,[\varnothing]} \rightarrow [-CH_2CH\,(OH)-]^{\varnothing} = K_5[-CH_2CH\,(OH)-][\varnothing]$$

$$r_6 = k_6K_1^{\frac{1}{2}}K_5\,[-CH_2CH\,(OH)-]\,P^{\frac{1}{2}}{}_{H2}[\varnothing]\,[*]$$

$$r_6 = k_6K_1^{\frac{1}{2}}K_5\,[EVOH]\,P^{\frac{1}{2}}{}_{H2}[\varnothing]\,[*]$$

II. Second hydrogen addition as the rate-limiting step (Step 7)

$$[-CH_2CH-]^* + [H^*] \xrightarrow[K_7]{} [-CH_2CH_2-]^* + [*]$$

$$r_7 = k_7\,[-CH_2CH-]^*\,[H^*]^2 \qquad \text{Eq. S4}$$

Using the equilibrium relationships for Steps 1, 5, and 6, the relevant surface coverages were expressed in terms of measurable reactant concentrations and substituted into the rate expression for Eq. S4.

$$K_6 = \frac{[-CH_2CH-]^*[H_2O^{\varnothing}]}{[-CH_2CH\,(OH)-]^{\varnothing}[H^*]} \rightarrow [-CH_2CH-]^* = \frac{K_6[-CH_2CH\,(OH)-]^{\varnothing}[H^*]}{[H_2O^{\varnothing}]}$$

$$K_5 = \frac{[-CH_2CH\,(OH)-]^{\varnothing}}{[-CH_2CH\,(OH)-]\,[\varnothing]} \rightarrow [-CH_2CH\,(OH)-]^{\varnothing} = K_5[-CH_2CH\,(OH)-][\varnothing]$$

$$r_7 = \frac{k_7K_6K_5K_1P_{H2}\,[EVOH][*]^2}{P_{H2O}}$$

13. References

(1) Bui, D.-P.; Wang, S.; Gomez, L. A.; Ul Karim, T.; Salas, T. S.; Abdolbaghi, S.; Nelson, K.; Lobban, L. L.; Maravelias, C. T.; Crossley, S. P. Self-Cleaning Catalysts Enable Recycling of Multilayered Plastic Films. *Green Chem.* 2025, *27* (44), 14076–14087. https://doi.org/10.1039/D5GC02739J.
